# Agrarian diet improves metabolic health in HIV-positive men with *Prevotella*-rich microbiomes: results from a randomized trial

**DOI:** 10.1128/msystems.01185-25

**Published:** 2025-11-26

**Authors:** John B. O'Connor, Jennifer Fouquier, Charles P. Neff, John D. Sterrett, Tyson Marden, Suzanne Fiorillo, Janet C. Siebert, Jennifer Schneider, Nichole Nusbacher, Amy T. Noe, Blair Fennimore, Janine Higgins, Thomas B. Campbell, Brent E. Palmer, Catherine Lozupone

**Affiliations:** 1 Department of Microbiology & Immunology, University of Colorado Anschutz 549224, Aurora, Colorado, USA; 2 Department of Biomedical Informatics, School of Medicine, University of Colorado Anschutz 727995, Aurora, Colorado, USA; 3 Department of Medicine, University of Colorado Anschutz 129263, Aurora, Colorado, USA; 4 Department of Integrated Physiology, University of Colorado Boulder 1877, Boulder, Colorado, USA; 5 CytoAnalytics, Denver, Colorado, USA; Katholieke Universiteit Leuven, Leuven, Belgium

**Keywords:** gut microbiome, metabolic syndrome, human immunodeficiency virus, metagenomics, inflammation, diet

## Abstract

This study aimed to assess the impact of a high-fiber/low-fat agrarian diet (AD) on inflammation and metabolic outcomes in HIV-positive men who have sex with men (MSM). Since the gut microbiomes of MSM resemble those of individuals in agrarian cultures, including being *Prevotella*-rich and *Bacteroides*-poor, we hypothesized that they would have particularly strong health benefits from consumption of a diet matched to their microbiome type. Sixty-six participants, including 36 HIV-positive MSM [HIV(+)MSM], 21 HIV-negative MSM, and 9 HIV-negative men who have sex with women, were randomized to either an AD or a high-fat/low-fiber western diet (WD) for 4 weeks. Plasma, fecal, and colonic biopsy samples were obtained. Metabolic and inflammatory markers were measured in plasma. 16S ribosomal RNA sequencing was performed on fecal and biopsy samples, and shotgun metagenomic sequencing was performed on fecal samples. The AD reduced plasma low-density lipoprotein cholesterol (LDL-C) in HIV(+)MSM, with median reductions of 0.4138 mmoL/L at 2 weeks and 0.2845 mmol/L at 4 weeks. Greater LDL-C reductions were predicted by *Prevotella*-rich/*Bacteroides*-poor microbiomes with increased starch utilization potential, emphasizing the importance of personalized microbiome-dietary matching. The AD also reduced T cell exhaustion and pro-inflammatory intermediate monocytes and altered host transcription in the colonic mucosa.

**IMPORTANCE:**

Our findings suggest tailoring diet interventions to baseline microbiome types can promote metabolic health in *Prevotella*-rich/*Bacteroides*-poor MSM, a significant portion of people living with HIV at risk for metabolic syndrome.

This study was registered at NCT02610374.

## INTRODUCTION

Thousands of human immunodeficiency virus type 1 (HIV-1) infections occur each day globally (1–3). Although antiretroviral therapy (ART) has improved the lifespan for people living with HIV (PLWH), they still face a higher incidence of metabolic and cardiovascular disease (CVD) (4–10). Gut microbiome dysbiosis and translocation of gut bacteria and bacterial products across intestinal epithelial mucosa contribute to chronic immune activation and inflammation, which are linked to CVD and other metabolic complications (6–8, 11–24). Characterizing the biological mechanisms underlying the gut microbiome and inflammatory and metabolic diseases in PLWH can improve treatment strategies.

HIV-associated gut microbiome dysbiosis, including lower diversity, increased Proteobacteria, and decreased commensal bacteria (14, 16, 17, 25–27), has been linked with metabolic co-morbidities and increased translocation in PLWH (27, 28). HIV-infected men in the United States and Europe also often have *Prevotella-*rich and *Bacteroides-*poor (PRBP) gut microbiomes, which occur more often in men who have sex with men (MSM) regardless of HIV infection status, and have been associated with factors such as having >3 sexual partners (16, 17, 28–36). Although studies have demonstrated that the PRBP microbiomes of MSM drive increased immune activation (37, 38), PRBP microbiomes are also characteristic of healthy HIV-negative individuals from non-Westernized cultures, including agrarian cultures in Africa, Amerindians, and the Hadza hunter-gatherers (36, 39–44). MSM PRBP microbiomes resemble those of individuals in non-Westernized cultures in a meta-analysis conducted with both 16S rRNA and shotgun metagenomic data, having high alpha diversity, high strain-level variation in *Prevotella* species, and similar co-occurring taxa (36, 45). Habitual intake of diets rich in carbohydrates, resistant starch, and fiber in individuals in Westernized cultures has been associated with PRBP microbiomes (40, 46–48). However, MSM exhibits PRBP microbiomes even when ingesting high-fat/low-fiber diets (16, 28, 29). Furthermore, PRBP microbiomes have also been observed to be enriched with obesity (49) and rheumatoid arthritis (RA) (50, 51). Thus, the health implications of PRBP microbiomes in MSM remain unclear.

By driving gut microbiome changes or changing the metabolic activity of the resident microbiome, diet can potentially reduce inflammation and metabolic comorbidity in PLWH (32). High-fat diets confer pro-inflammatory responses and accelerated disease progression in macaques infected with simian immunodeficiency virus (SIV) (52). Furthermore, murine models have shown *Bacteroides uniformis*, which is depleted in MSM, to ameliorate metabolic and immune dysfunction induced by a high-fat diet (12, 53–55), suggesting diets lower in fat may be particularly beneficial to HIV-positive MSM. Many studies have also indicated that having a PRBP microbiome can be beneficial in the context of a high-fiber diet and detrimental in a high-fat/low-fiber diet. For instance, PRBP microbiomes in non-MSM populations have been associated with favorable metabolic health parameters, such as improved postprandial glucose metabolism (56) or lower levels of low-density lipoprotein cholesterol (LDL-C) (48). In high-fiber diet interventions, high *Prevotella* at baseline has been associated with greater weight loss in obese individuals (57–59), improved glucose metabolism (60), and a greater decrease in total cholesterol, LDL-C, and triglycerides in HIV-negative men with metabolic syndrome (metS) (61). Other studies have suggested that, in the context of a high-fat and/or low-fiber diet, PRBP microbiomes can promote obesity, poor glycemic control, and lipid profiles contributing to metS (49, 62–64). Mechanistically, beneficial effects have been linked with the production of metabolites like succinate from dietary fiber (65), and negative effects have been linked with branched-chain amino acids (BCAAs) production in high-fat diet contexts (62). Taken together, these studies suggest that ingestion of a high-fat/low-fiber western diet (WD) with a PRBP microbiome may result in a diet/microbiome mismatch, contributing to inflammatory and metabolic diseases. We thus hypothesized that intake of an AD that matches the PRBP microbiome common in HIV positive and negative MSM can help mitigate HIV-associated metabolic comorbidity.

This study assessed the effect of short-term diet modification on inflammatory/metabolic-disease markers in HIV-positive MSM on ART and HIV-negative controls. Participants consumed either an AD or a WD, which mimicked a typical U.S. diet. Our findings suggest that tailoring diet interventions to baseline microbiome types can promote metabolic health in *Prevotella*-rich/*Bacteroides*-poor MSM.

## RESULTS

### Study cohorts were comparable at baseline

Sixty-six individuals were enrolled, including 36 (54.5%) HIV-positive MSM [HIV(+)MSM], 21 (31.8%) HIV-negative MSM [HIV(−)MSM], and 9 (13.6%) HIV-negative men who have sex with women (MSW) [HIV(−)MSW] (Table 1). Six individuals were lost to follow-up, but baseline values were included in the analysis. HIV(+)MSM were older than HIV(−)MSW (*P*adj = 0.010). Most HIV(+)MSM were using two nucleoside reverse transcriptase inhibitors (NRTIs) with an integrase strand transfer inhibitor (INSTI; 67.6%) or non-nucleoside reverse transcriptase inhibitors (NNRTIs; 17.6%).

**TABLE 1:** Study population^*a*^

Cohort	HIV(+)MSM	HIV(−)MSM	HIV(−)MSW
Sample number	36	21	9
Age (median [IQR])	46.03 (33.11–54.57)	39.57 (30.24–46.96)	29.38 (24.07–34.57)
BMI (median [IQR])	25.90 (23.30–28.40)	25.60 (24.00–27.00)	26.30 (25.60–27.00)
Diet			
Agrarian (count [%])	18 (50.0%)	13 (61.9%)	4 (44.4%)
Western (count [%])	18 (50.0%)	8 (38.1%)	5 (55.6%)
Distance to standard agrarian (median [IQR])	1.63 (1.52–1.87)	1.68 (1.37–1.8)	1.59 (1.51–1.69)
Metabolic markers			
HDL-C (mg/dL) (median [IQR])	40.00 (34.50–52.00)	49.00 (39.00–55.00)	42.00 (34.00–48.00)
LDL-C (mg/dL) (median [IQR])	102.00 (83.00–117.50)	94.00 (78.00–121.00)	80.00 (72.00–112.00)
Triglycerides (mg/dL) (median [IQR])	119.00 (93.00–203.00)	83.00 (63.00–181.00)	99.00 (84.00–130.00)
HOMA-IR (median [IQR])	1.44 (0.78-2.32)	0.92 (0.76-2.53)	1.06 (0.64-1.17)
Inflammatory markers			
LBP (μg/μL) (median [IQR])	34.96 (33.03–36.54)	35.6 (33.73–36.64)	35.03 (32.03–35.58)
IL-6 (pg/mL)(median [IQR])	3.95 (1.91–5.50)	2.81 (1.73–3.41)	2.75 (2.32–8.02)
CRP (mg/L)(median [IQR])	3.21 (2.3–5.22)	2.68 (1.95–4.07)	2.39 (1.22–3.42)
Alpha diversity			
Fecal Shannon (median [IQR])	5.54 (5.05–5.83)	5.95 (5.60–6.45)	5.41 (5.12–5.83)
Fecal Faith (median [IQR])	18.91 (15.58–21.89)	22.33 (17.74–26.90)	21.78 (19.00–26.07)
Biopsy Shannon (median [IQR])	5.44 (4.69–5.70)	5.86 (5.37–6.16)	5.72 (5.63–5.85)
Biopsy Faith (median [IQR])	23.94 (15.26–33.22)	19.48 (14.31–22.37)	26.82 (14.90–35.39)
T cell percentages			
CD4+ T cells/mL (median [IQR])	628 (514.50–945.25)	NA	NA
CD8+ T cells/mL (median [IQR])	709 (590.00–925.50)	NA	NA
Antiretroviral therapy combinations (*N* = 34**)**			
2 NRTIs and INSTI	23 (67.6%)	NA	NA
2 NRTIs and NNRTI	6 (17.6 %)	NA	NA
Other	5 (14.7%)	NA	NA

^*a*^ Baseline data split by HIV and MSM status with data presented as N, median (interquartile range), or *n* (%), unless otherwise stated. HIV = human immunodeficiency virus, MSM = men who have sex with men, MSW = men who have sex with women, BMI = body mass index, IQR = inter-quartile range, LDL-C = low-density lipoprotein cholesterol, HDL-C = high-density lipoprotein cholesterol, HOMA-IR = Homeostatic model assessment for insulin resistance, LBP = lipopolysaccharide binding protein, IL-6 = interleukin-6, CRP = C-reactive protein, NRTI = nucleoside reverse transcriptase inhibitor, INSTI = integrase strand transfer inhibitor, NNRTI = non-nucleoside reverse transcriptase inhibitor. NA indicates measures not applicable to the HIV negative cohorts.

### AD included expected dietary changes

After randomization to AD or WD, participants consumed 2 weeks of prepared meals followed by 2 weeks of guided self-feeding. The WD arm had a target content of fat, fiber, sugar, sodium, and carbohydrates approximating typical diets in the United States and other Western countries (Table 2). The AD mimicked diets of agrarian African cultures, which are higher in total fiber and lower in fat, protein, sugar, and sodium (66). To avoid negative medical consequences on an AD, instead of providing lower protein food items, high protein foods commonly consumed in Africa were provided, such as lean whole meats and eggs. Foods high in resistant starch (RS) and yams were used to mimic cold maize porridge and cassava, agrarian African staples, so that a suitable mixture of soluble and insoluble fiber was provided during AD consumption (67). Thus, the intervention diet was similar in composition to the agrarian African diet in having a high complex-to-simple carbohydrate ratio, but palatable to our study population (68–70).

**TABLE 2:** Standard diet compositions^*a*^

Diet composition	Agrarian T2	Agrarian T3	Western T2	Western T3
Fat (%)	15 ± 0.0002	21.3 ± 1. 2	30 ± 0.0002	38.6 ± 0.8
Saturated fat (%)	3.2 ± 0.0002	3.5 ± 0.0002	10 ± 0.0009	10 ± 0.002
Carbohydrate (%)	70 ± 0.0006	55.4 ± 1.2	55 ± 0.0003	45.9 ± 0.9
Protein (%)	15 ± 0.0002	21.9 ± 0.8	15 ± 0.00017	15.4 ± 2.9
Sodium (mg/day)	1,500 ± 9.2	1,053 ± 128	3,500 ± 12.75	3,985 ± 199
Fiber (g/1,000 kcal)	22 ± 0.1	21.9 ± 0.8	9 ± 0.07	6.3 ± 0.3
Soluble fiber (g/1,000 kcal)	5.5 ± 0.2	6.4 ± 0.4	2.3 ± 0.1	2.3 ± 0.01
Insoluble fiber (g/100 kcal)	18 ± 0.7	14 ± 0.05	5.0 ± 0.6	4.6 ± 0.02
Pectins (g/100 kcal)	4.5 ± 0.2	2.1 ± 0.01	1.0 ± 0.2	0.9 ± 0.01
Sugar (g/1,000 kcal)	30 ± 0.1	27.1 ± 2.1	57 ± 0.2	47.5 ± 1.

^*a*^ Median and interquartile range values of dietary macronutrients in grams per 1,000 kcal stratified by diet and timepoint as determined by participant self-reporting.

**Fig 1:**
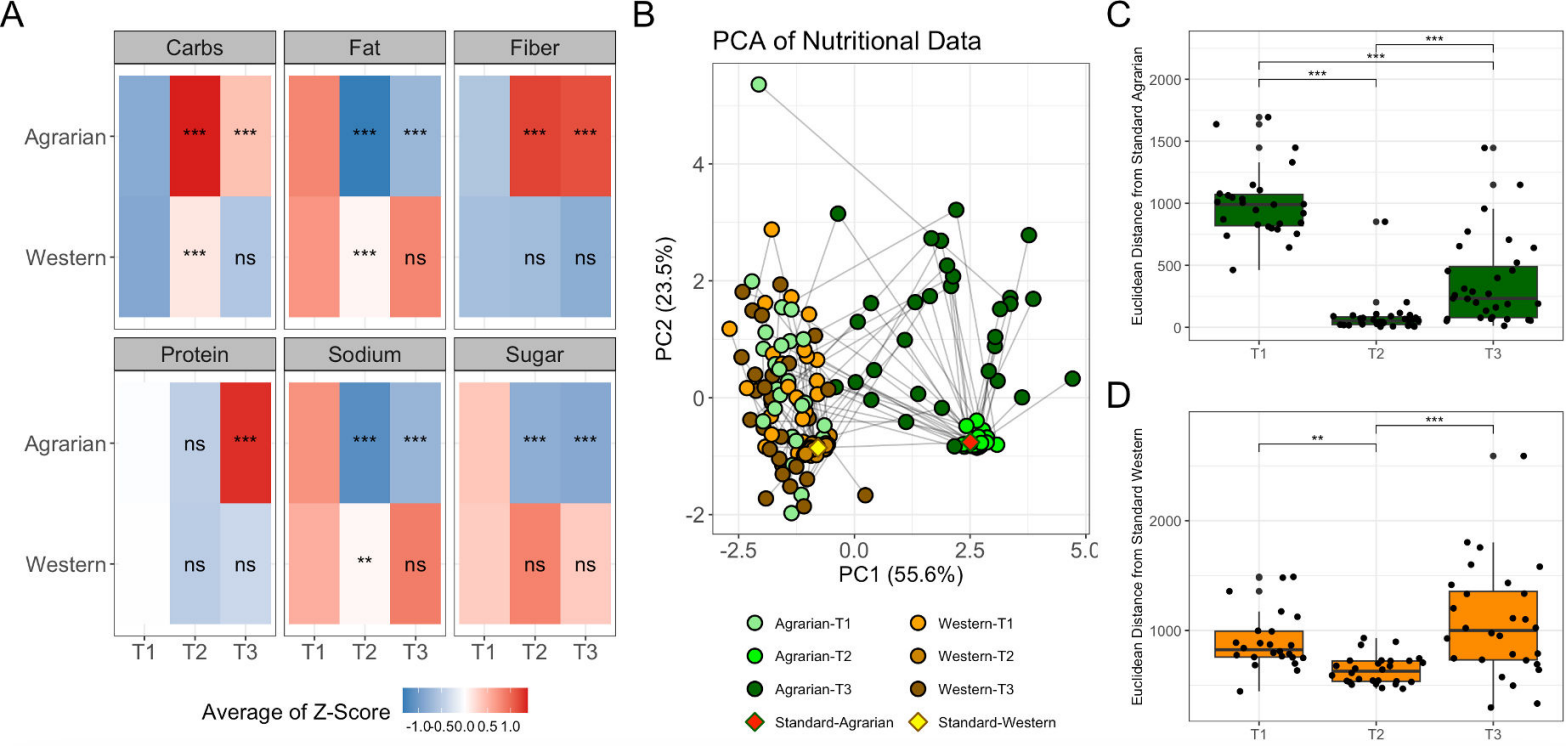
AD intervention associated with dietary change toward target macronutrient intake. (**A**) Heatmap of dietary components at baseline (T1), 2 weeks (T2), and 4 weeks (T3) for individuals on an agrarian or western diet intervention. The heatmap is colored using the average log-midpoint transformed values for carbohydrates, fat, fiber, protein, sodium, and sugar, normalized to 1,000 kcals. Significance for dietary composition levels between timepoints was tested using a paired nonparametric Friedman test with a Bonferroni correction for multiple comparisons to compare both T2 and T3 with T1/baseline. (**B**) Principal component analysis plot from a Euclidean distance matrix of macronutrient totals estimated from reported nutritional data, including protein, fat, carbohydrates, fiber, sodium, and sugar. Points are colored by diet intervention arm and time point. The target macronutrient compositions (averages of T2) are also plotted as “Standard-Agrarian” and “Standard-Western.” (**C, D**) Distances from the standard target diet for those on the agrarian (**C**) Western (**D**) diets with brackets indicating significance determined by the Wilcoxon-signed rank test. *P < 0.05, **P < 0.01, ***P < 0.001. PCA = principal components analysis, PC = principal component, ns = not significant.

Diet was assessed at baseline (T1) using the Diet History Questionnaire II (DHQ2) (71). Diet compliance after the first 2 weeks (T2) of prepared food was monitored during meal pickup, when uneaten or extra food consumed was recorded. Compliance after the second 2-week period of self-prepared diet (T3) from a set menu was monitored by 24 h food recall. Individuals on the AD consumed elevated carbohydrates and fiber and decreased fat, sodium, and sugar at T2 compared with baseline and, to a lesser extent, at T3 (Fig. 1A). Individuals on the WD consumed slightly elevated carbohydrates, reduced fat, and reduced sodium at T2 compared with baseline and had no significant changes from baseline at T3. Those on the WD still had higher fat (*P* = 2.98 × 10^-10^) and lower carbohydrates (*P* = 9.07 × 10^−11^) than those on the AD at T2.

A principal component analysis (PCA) plot based on total macronutrients indicates T1 nutritional intake was consistent with the WD target (Fig. 1B). AD conferred bigger disturbances from baseline at T2 (*P*=1.05 × 10^-8^) and T3 (*P* = 0.006) compared with WD as measured by Euclidean distance from baseline nutritional intake. Reduced Euclidean distance to the target diet compositions at T2 and to a lesser extent T3, with variability lower in T2, indicated there was optimal compliance at T2, but diets were still maintained at T3 (Fig. 1C and D).

### AD reduced lipoprotein cholesterol

Fasting metabolic markers, including triglycerides, Homeostatic Model Assessment for Insulin Resistance (HOMA-IR; calculated using fasting blood glucose and insulin levels), LDL-C, and high-density lipoprotein cholesterol (HDL-C), were evaluated. There were trends toward lower HDL-C and higher LDL-C, triglycerides, and HOMA-IR in the HIV(+)MSM at baseline, which is consistent with past studies (72–74). Metabolic health measures did not differ across ART regimens.

**Fig 2:**
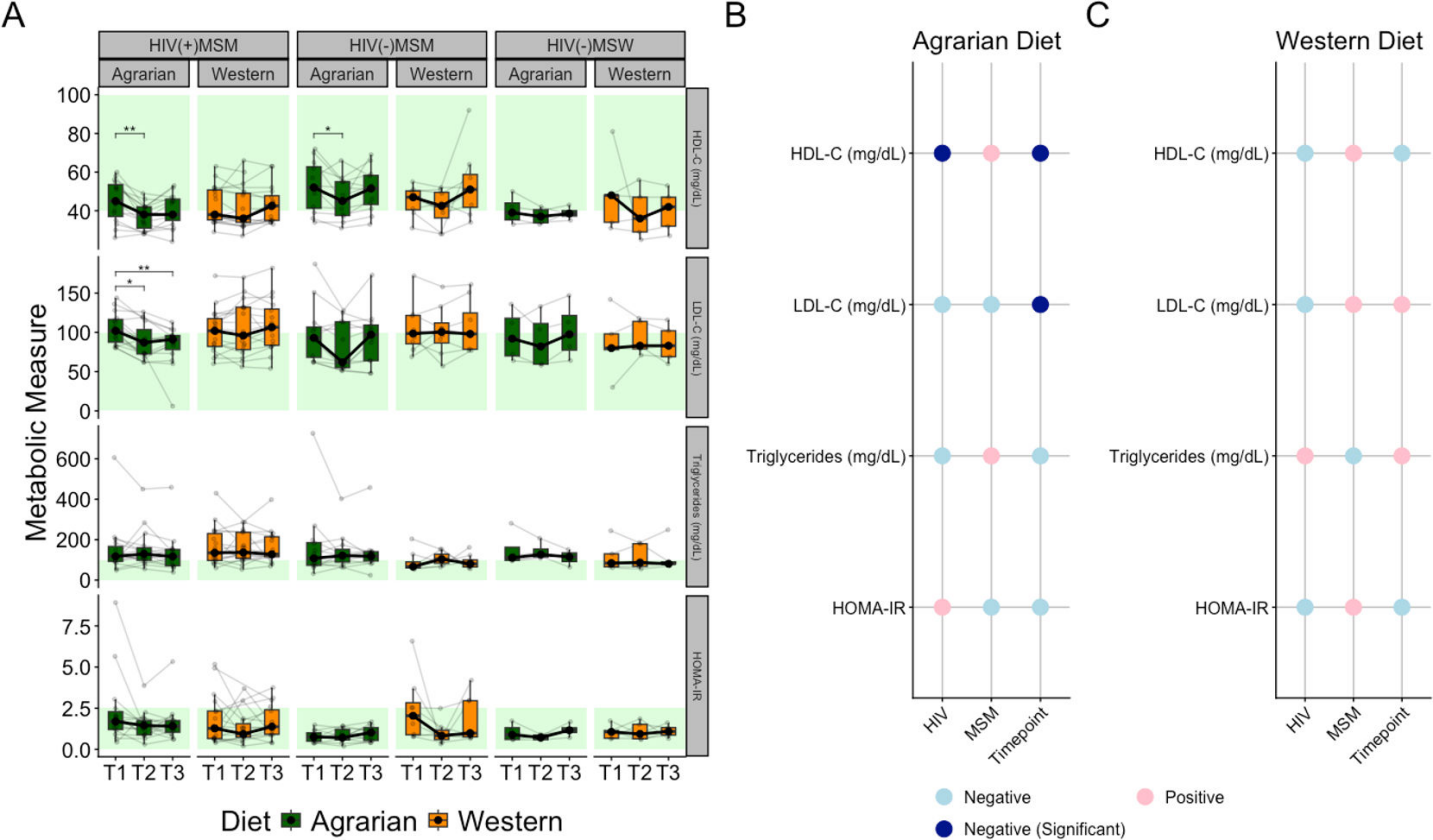
Reduced plasma cholesterol levels in MSM participants on the agrarian diet. (**A**) Box plots overlaid with spaghetti plots of metabolic health markers colored by diet with brackets indicating significance as determined by Friedman test with Bonferroni multiple comparisons correction, thin lines and dots represent values from individual participants while thick black lines and dots represent median values; *P ≤ 0.05, **P ≤ 0.01. Healthy ranges for each measure by previously established thresholds are indicated with green shading (75–77). Panels **B** and **C** show coefficients of linear mixed-effects models relating metabolic health markers to MSM status, HIV infection status, and time point timepoint (continuous variable representing time on the diet intervention) in (**B**) those on the AD and (**C**) those on the WD. Red indicates a positive relationship, whereas blue indicates negative. Darker blue in panel B represents statistical significance at *P* < 0.05. *P*-values determined by analysis of variance (ANOVA) of the full model [metabolic marker ~ HIV + MSM + (1|StudyID)] vs model removing the predictor of interest. HIV = human immunodeficiency virus, MSM = men who have sex with men, MSW = men who have sex with women, HDL-C = high-density lipoprotein cholesterol, LDL-C = low-density lipoprotein cholesterol, and HOMA-IR = homeostatic model assessment for insulin resistance.

Both MSM AD groups had reduced HDL-C at T2 that was not sustained at T3 (Fig. 2A). In HIV(+)MSM, AD reduced LDL-C at T2 and T3. No significant changes occurred in HOMA-IR or triglycerides. Linear mixed-effects models (LMEMs) confirmed that LDL-C and HDL-C were negatively related to time on the AD, with HDL-C also being negatively related to HIV infection, as previously reported (Fig. 2B) (73). No relationships with time were observed on the WD (Fig. 2C). LMEMs revealed HDL-C (*P*adj = 9.42 × 10^−5^) and LDL-C (*P*adj = 4.47 × 10^−3^) increased with distance to standard AD, indicating that they reduced with more agrarian diets.

Participants with metabolic measures in the healthy range at baseline had the highest probability of being in the healthy range after AD (Fig. 2A; Fig. S1). Furthermore, when analyses were repeated only on individuals with metabolic measures in the unhealthy range at baseline, only LDL-C reduction from T1 to T3 (*P*adj = 0.014) was significant, indicating unhealthy low HDL-C levels were not exacerbated by either diet.

### AD impact on inflammatory markers

We quantified inflammation with C-reactive protein (CRP) and interleukin-6 (IL-6), and intestinal permeability with lipopolysaccharide binding protein (LBP). CRP and IL-6 trended higher in HIV(+)MSM as expected based on prior studies (Table 1) (78). IL-6 decreased from T1 to T3 in HIV(+)MSM on the AD, but with no statistical significance (*P*adj = 0.06) (Fig. S2A). LBP was reduced from T1 to T2 in the WD in HIV(+)MSM (*P*adj = 0.023). LMEMs identified no significant relationships between inflammatory markers and time, HIV infection status, or MSM status when stratifying by diet (Fig. S2B and C), and inflammatory measures did not correlate with distance to the standard AD. Inflammatory marker changes also depended on baseline values (Fig. S2D), with higher baseline values correlating with greater reduction. In samples with high baseline values (top 50th percentile), a significant reduction in IL-6 at T2 was observed on the AD (*P*adj = 0.008) that was somewhat sustained at T3 (*P*adj = 0.058)(Fig. S2D and E). A significant IL-6 reduction was not observed when performing the same analysis on the WD arm, suggesting that this is not simply an artifact of regression to the mean.

### AD altered peripheral monocyte differentiation and reduced T-cell exhaustion

**Fig 3:**
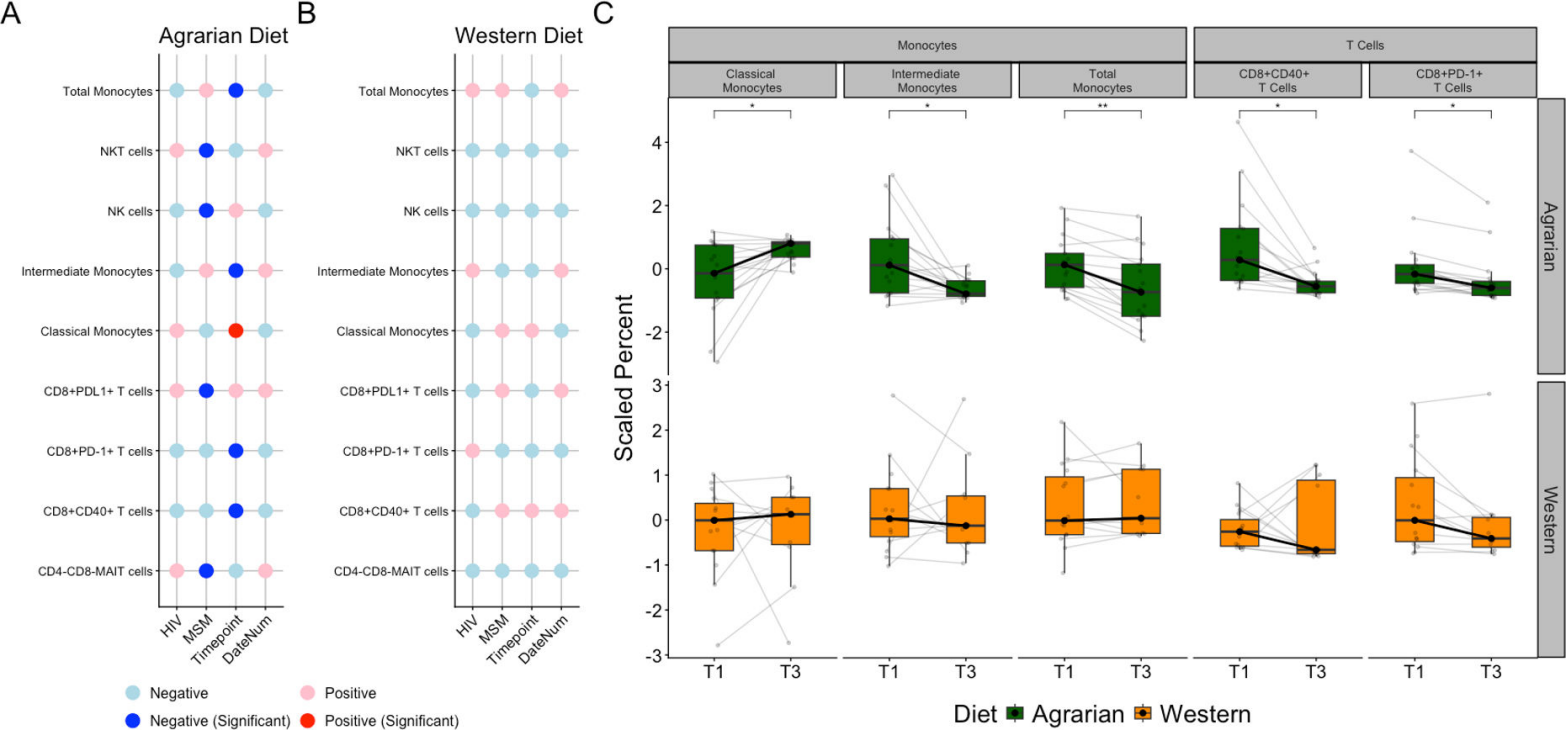
Blood immune cell populations change with diet intervention. (**A and B**) Coefficients of linear mixed-effects models (LMEMs) relating blood immune cells to MSM status, HIV infection status, and timepoint timepoint (continuous variable representing time on the diet intervention) in (**A**) those on the agrarian diet and (**B**) those on the western diet. Red indicates a positive relationship, and blue indicates the opposite. Dark red and blue in panel A represent significance at *P* < 0.05. *P*-values determined by analysis of variance (ANOVA) of the full model [immune cell population ~ HIV + MSM+(1|StudyID)] vs a model removing the predictor of interest. Cells displayed only include those with some significant relationship to the AD. There were no cell populations significant with timepoint (i.e., before and after diet intervention) on the western diet (WD) (**C**) Box plots overlaid with spaghetti plots of blood immune cells colored by diet, thin lines and dots represent values from the individual participants while thick black lines and dots represent median values; asterisks indicate *P*-values determined by ANOVA of LMEMs with FDR correction for multiple comparisons; NKT = natural killer T, NK = natural killer, MAIT = mucosal-associated invariant T, HIV = human immunodeficiency virus, and MSM = men who have sex with men.

Cytometry by time-of-flight (CyTOF) was performed on peripheral blood mononuclear cells (PBMCs) collected at T1 and T3. The monoclonal antibody (mAb) panel (Table S1) targeted diverse immune populations, focusing on CD4+and CD8+ T cells, but also characterizing monocytes, macrophages, dendritic cells, B cells, natural killer (NK) cells, NK T cells, and mucosal-associated invariant T cells (MAIT). A representative gating strategy is shown in Fig. S3. Permutational multivariate analysis of variance (PERMANOVA) of the CyTOF data revealed that the date of the run contributed significantly to the results (*R*^2^ = 0.042, *P* = 0.008), indicating batch effects. Thus, a continuous time variable (DateNum) for the day of the run was incorporated into all models. When stratifying by cohort, HIV(+)MSM on the AD had significantly reduced CD8+ PD1+ T cells, and HIV(−)MSM on the AD had significantly reduced CD4+ CD38+ HLA-DR+ T cells, with no change on WD (Fig. S4). The other groups exhibited similar trends but lacked significance, possibly due to the limited sample number. In unstratified analyses, LMEMs revealed a significant increase in phagocytic CD14 +CD16 cells (classical monocytes) and a decrease in CD14+ cells (total monocytes), CD14+ CD16+ cells (intermediate monocytes), CD8+ PD-1+ T cells, and CD8+ CD40+ T cells in those on AD but not on WD (Fig. 3).

### Baseline fecal microbiome predictive of AD LDL-C response

**Fig 4:**
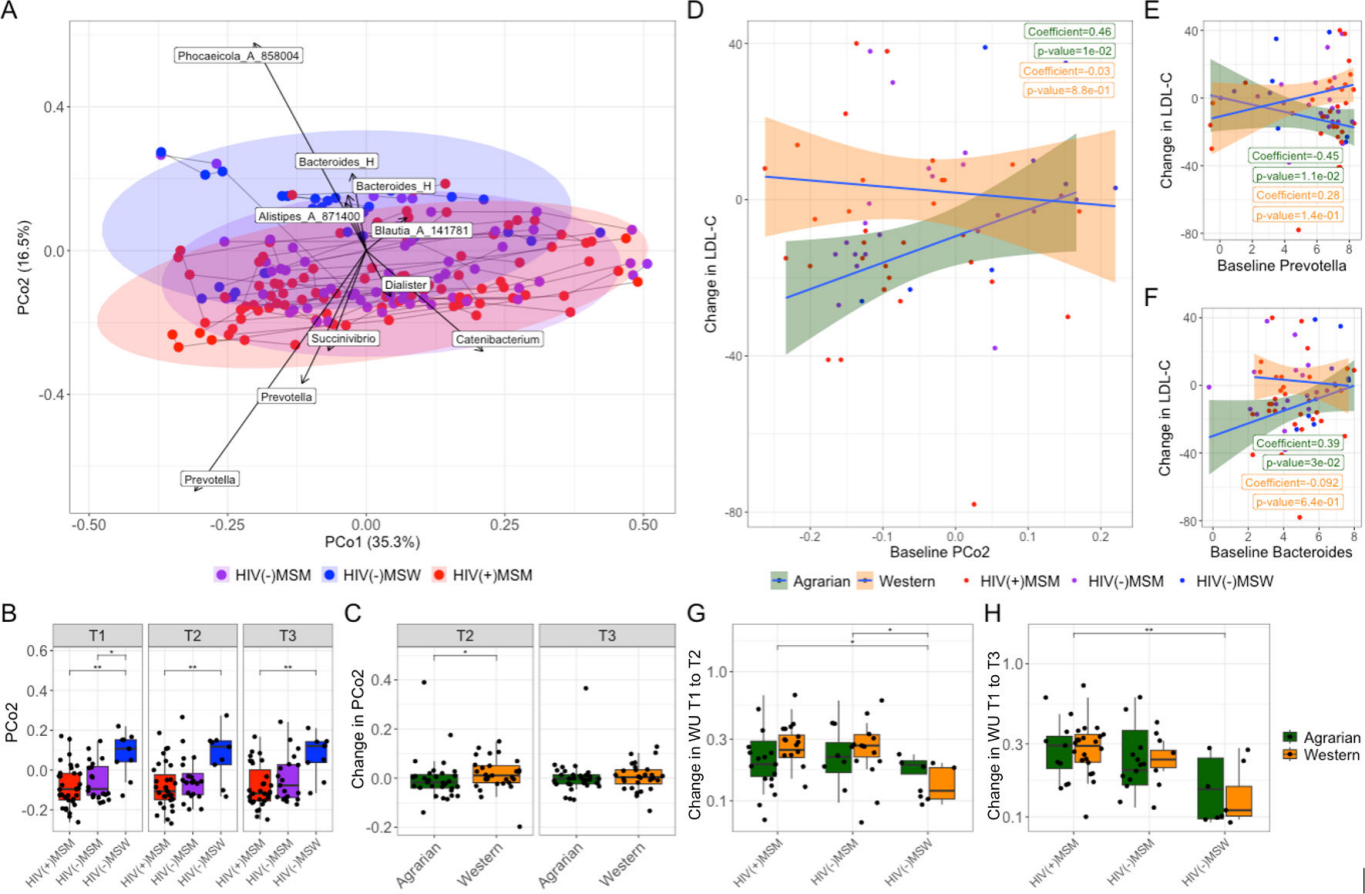
Fecal microbiome beta diversity analysis. (**A**) Principal coordinates analysis (PCoA) of weighted UniFrac values of fecal 16S rRNA data. Points are colored by cohort. Samples from the same individual over time (T1: baseline, T2: after 2 weeks of prepared diets, T3: after an additional 2 weeks of self-prepared meals) are joined with a gray line. Bacterial genera are plotted in the same space using the biplots functionality of QIIME 2 (81). The length of the arrows indicates the strength of the relationship with PCoAs 1 and 2. (**B**) PCo2 values for the three cohorts at T1, T2, and T3. Statistical significance across three groups was determined by Kruskal-Wallis and pairwise comparisons made using Dunn’s *post hoc* test. (**C**) Changes in PCoA 2 from T1 to T2 (left panel) and T1 to T3 (right panel); statistical significance determined by the Wilcoxon signed-rank test. (**D,E,F**) Scatter plot of change in LDL-C from T1 to T3 versus values of (**D**) PCo2, (**E**) center-log ratio (CLR) transformed *Prevotella* abundance, and (**F**) CLR transformed *Bacteroides* abundance at baseline with Spearman correlations and *P* values of the two separate diets. Weighted UniFrac distances from baseline to (**G**) T2 and (**H**) T3; groupwise statistics determined by Kruskal-Wallis with Dunn’s *post hoc* test; *P ≤ 0.05, **P ≤ 0.01, PCo = principal coordinates analysis axis, MSM = men who have sex with men, MSW = men who have sex with women, HIV = human immunodeficiency virus, and LDL-C = low density lipoprotein cholesterol.

16S ribosomal RNA (rRNA)-targeted sequencing was used to characterize the fecal microbiome. PERMANOVA by sequencing run of weighted UniFrac beta diversity indicated no batch effects. Principal coordinate analysis (PCoA) of weighted UniFrac distances (79) indicated that fecal microbiomes separated based on *Prevotella* relative abundance versus *Bacteroides* and *Phocaeicola* (formerly *Bacteroides*) along PCo2 (Fig. 4A) (79). As previously reported, MSM had more PRBP microbiomes (lower PCo2 values) than MSW (Fig. 4A and B) (28, 29). ANCOM-BC indicated baseline compositional differences with MSM status, including depleted *Bacteroides* (Fig. S5A; Table S2) (80). PERMANOVA indicated community difference by MSM status (*R*^2^ = 0.044, *P* = 0.001), with no significance by diet or HIV status. However, ANCOM-BC did detect significantly different genera between HIV(+)MSM and HIV(−)MSM at baseline (Fig. S5B; Table S2), with HIV(+)MSM also having somewhat reduced Shannon diversity (*P* = 0.069).

No AD-associated changes in the fecal genera were observed when stratifying by cohort (Table S3). In unstratified analyses, two genera, BX12 from the *Anaerovoracaceae* family and *Paramuribaculum*, showed a positive relationship with time on AD (Table S4). Relative abundances of the most abundant fecal genera are displayed in Fig. S3. Samples clustered by individual in PCoA (*R*^2^ = 0.730, *P* = 0.001), consistent with studies showing high interpersonal variation (46, 82–84). Weighted UniFrac distances from baseline did not differ by diet (Fig. 4G and H). MSM microbiomes had larger changes from baseline compared with MSW regardless of diet, suggesting higher volatility (Fig. 4G and H). There was an AD-associated reduction in PCo2 and a WD-associated increase in PCo2, which separated individuals based on *Prevotella* and *Bacteroides*/*Phocaeicola*, from T1 to T2, suggesting dietary influence on the PRBP microbiome type upon initial intervention, with significance lost from T1 to T3 (Fig. 4C).

We compared baseline PCO2 to changes in metabolic health markers. Strikingly, lower baseline PCO2 values (i.e., more PRBP microbiomes) correlated with greater LDL-C reductions from T1 to T3 on the AD (Spearman *P* = 0.010) (Fig. 4D), whereas the opposite was true for the WD, although not significant. The same was true for elevated baseline *Prevotella* (Spearman *P* = 0.011) and reduced baseline *Bacteroides* (Spearman *P* = 0.030) (Fig. 4E and F).

To investigate genera related to reduced LDL-C, we used EXPLANA, a software workflow for exploratory analysis with mixed effects random forest (MERF) (85). Inputting 490 bacterial genera, HIV-infection status, MSM status, and time point as predictors of LDL-C measurements over the three time points, we could explain 9.2% of the variation in LDL-C values, with 12 features selected as significantly important using the Boruta method (Fig. S6A, left panel 1) (86). Using EXPLANA, we also calculated deltas between all pairs of timepoints in both the outcome (LDL-C) and predictors prior to MERF. When analyzing whether all pairwise changes between the three time points could predict the change in LDL-C between the same time points, a 19.1% variation in LDL-C delta values was explained, and 10 features were selected as important (Fig. S5B and S6A, right panel). The same feature selection method on the WD group showed no strong predictive capabilities for LDL-C. *Collinsella* and *Negativibacillus* were identified as important in both models (Fig. S6A). Decreasing levels of *Collinsella* and *Parabacteroides* between time points and increasing *Bilophila* were the top features related to the reduction in LDL-C between time points (Fig. S6C).

**Fig 5:**
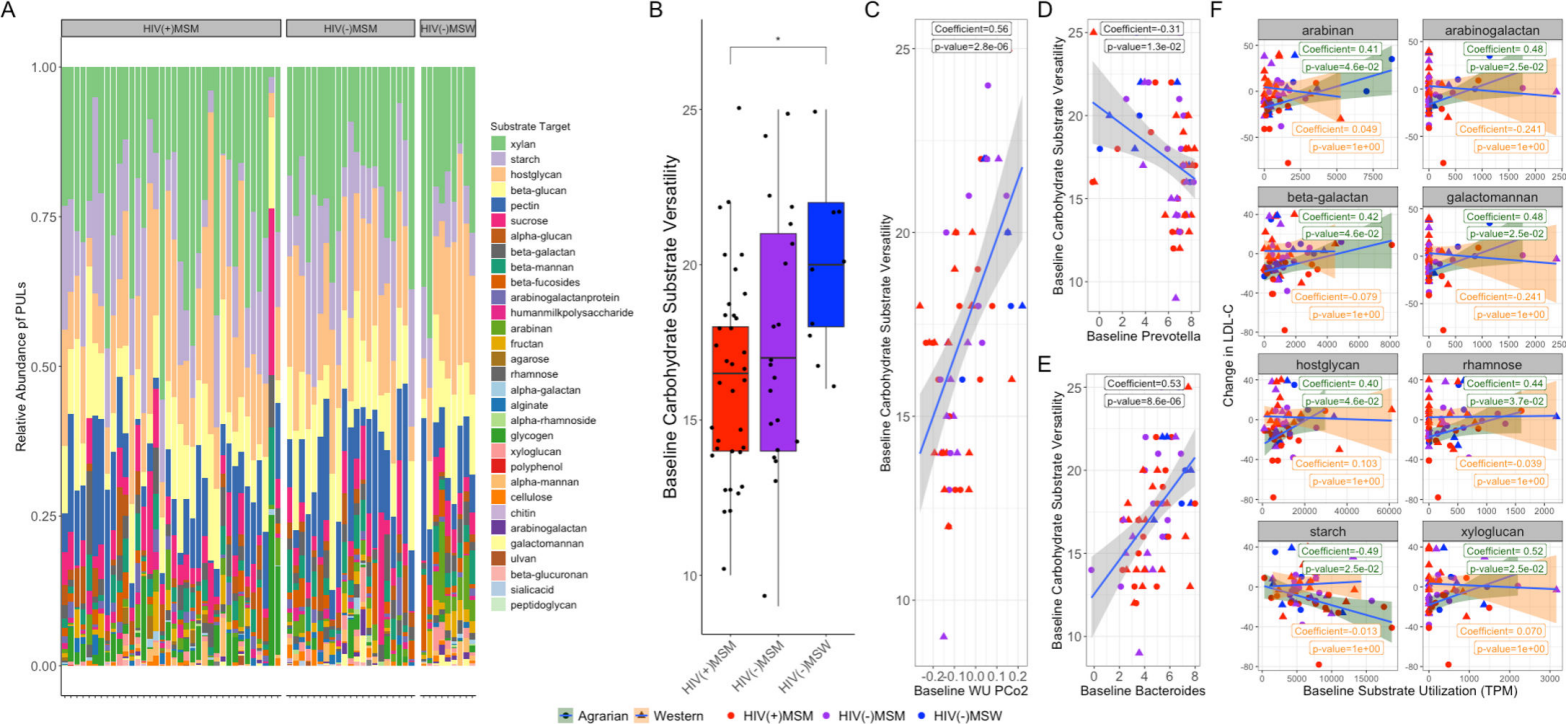
PRBP microbiomes are less functionally versatile and target different carbohydrate substrates. (**A**) Barplot of relative polysaccharide utilization loci (PULs) colored by substrate target and split by cohort at baseline, as determined by analysis of fecal shotgun metagenomic data with dbCAN3. (**B**) Boxplot of baseline carbohydrate substrate versatility (# of substrates based on PULs in each sample) split by group with brackets indicating significance as determined by Kruskal–Wallis and Dunn’s *post hoc* test; *P ≤ .05. (**C to E**) Scatter plots of baseline carbohydrate substrate versatility versus (**C**) baseline weighted UniFrac PC axis 2, (**D**) baseline CLR-transformed *Prevotella* abundance, and (**E**) baseline CLR-transformed *Bacteroides* abundance. (**F**) Scatter plots of change in LDL-C versus total PULs targeting specific substrates at baseline, colored by group, with fits colored by diet; statistics indicate Spearman correlations for all participants (black), participants on agrarian diets (green), and participants on Western diet (orange).

Shotgun metagenomic sequencing of baseline fecal samples was performed, and dbCAN3 (87) was used to predict the substrates of encoded polysaccharide utilization loci (PULs). Microbial PULs targeted a wide variety of substrates, with xylan, starch, and host glycans the most common (Fig. 5A). In LMEMs, comparing substrates to both MSM and HIV status, MSM status was associated with reduced arabinan targeting.

HIV(+)MSM microbiomes had lower carbohydrate substrate versatility, with PULs targeting fewer substrates than more functionally capable HIV(−)MSW microbiomes (*P*adj = 0.028) (Fig. 5B). *Prevotella* richness and *Bacteroides* depletion correlated with less versatility (Fig. 5C through E). PRBP microbiomes were associated with reduced utilization potential of 14 substrates, including arabinan, alpha-mannan, arabinogalactan, galactomannan, rhamnose, host glycans, beta-galactan, xyloglucan, fructan, beta-glucan, beta-mannan, sucrose, alpha-galactan, and beta-glucuronan, but showed increased starch utilization potential. Of these, 15 total substrates related to PRBP microbiomes, seven correlated negatively with the AD-associated drop in LDL-C (Fig. 5F), with only starch utilization potential correlating with a better response.

### AD altered host transcription

Changes in the microbiome, immune cells, and host transcription were assessed on 54 colonic biopsy samples, which were only collected at T1 and T3. Mucosal microbiome composition differed by HIV status but not MSM status (Fig. S7A; Table S5).AD-associated changes in low-abundance genera were observed, including reductions in *Peptoniphilus_A*, *Onthomonas*, *VUNA01,* and increases in *Pseudobutyricicoccus* in HIV(+)MSM, as well as overall increases in *Pseudobutyricicoccus* and *COE1* and reductions in *VUNa01* and *RUG13077* on the AD as determined by unstratified analysis (Fig. S7B and C; Tables S6 and S7). PERMANOVA indicated no statistically significant batch effects by sequencing run. When using the date of the run to account for CyTOF batch effects, no changes in biopsy immune cells were observed when stratifying by cohort and diet. Stratifying by diet alone, LMEMs showed no significant relationships with time on AD, whereas WD was associated with select immune cell changes (Fig. S8A and B). Biopsy immune cells showed no relationship with LDL-C (Fig. S8C).

**Fig 6:**
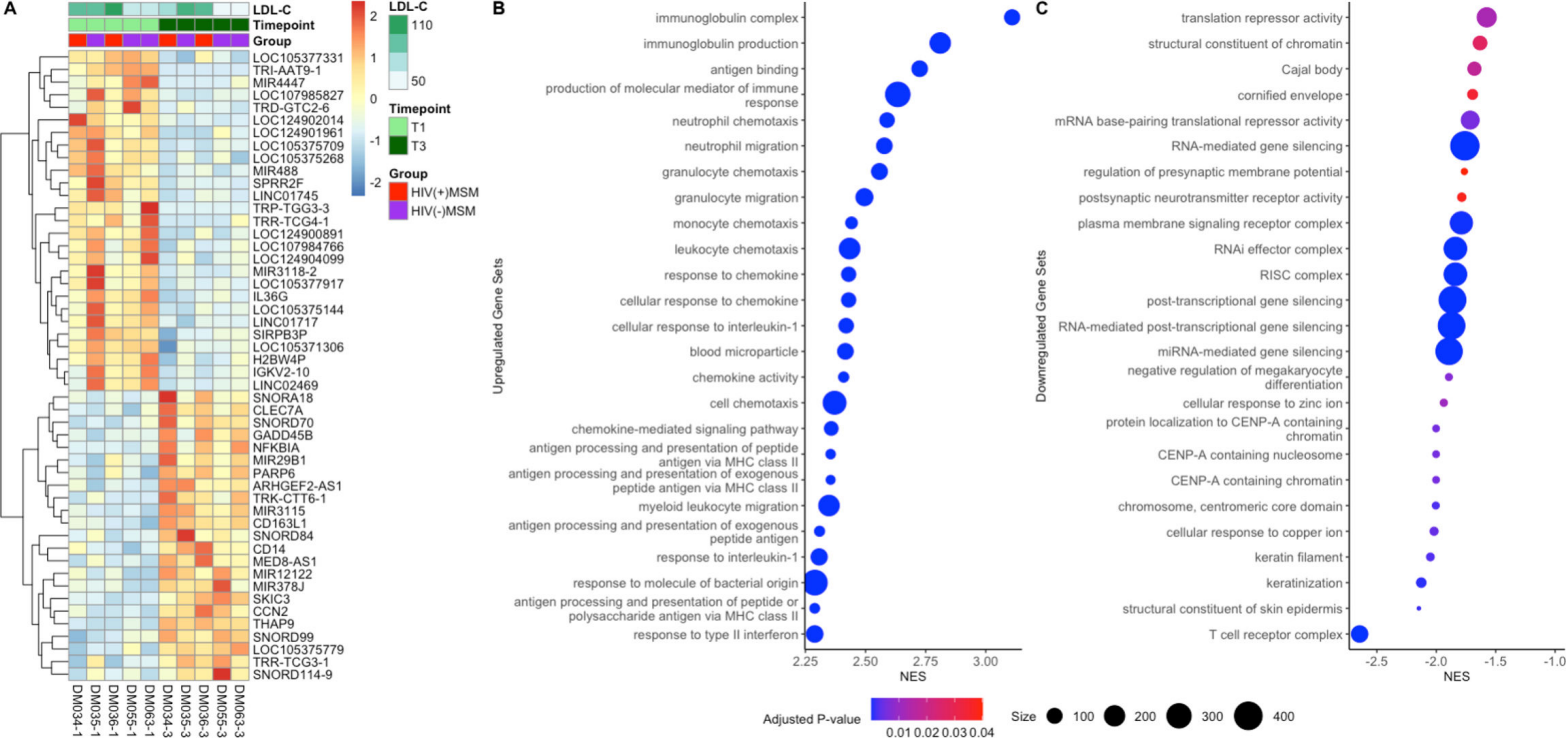
Biopsy transcriptome differences by HIV and time on AD. (**A**) Heatmap of expression of the 50 most differentially expressed gene transcripts (*y*-axis) in participants (x-axis) before and after 4 weeks on AD as determined by Limma, with gene transcripts hierarchically clustered according to Euclidean distance and samples ordered by StudyID and time point, and annotated with LDL-C values and group. (**B**) Twenty-four most enriched gene sets, and (**C**) 24 most depleted gene sets as determined by the lowest FDR-adjusted *P*-values obtained by gene set enrichment analysis (all < 0.05). The number of genes in a gene set (size) is indicated by the size of the dots. Higher normalized enrichment scores (NES) (*x*-axis) indicate enrichment at the later time point, whereas lower NES scores indicate depletion. LDL-C = low-density lipoprotein cholesterol, HIV = human immunodeficiency virus, and NES = normalized enrichment score.

In 5 MSM individuals on the AD with high compliance at T3, RNA sequencing was performed on the biopsy samples, and limma was used to identify differential expression between T1 and T3 (88). No significant changes in host transcription were found within the HIV(+)MSM and HIV(−)MSM cohorts, which were limited in the sample number. When including all five samples on AD and accounting for HIV status, TRI-AAT9-1, a tRNA gene, decreased with the AD. Figure 6A shows the expression of the 50 most differentially expressed genes. Gene set enrichment analysis (GSEA) revealed increased expression of genes related to immune trafficking (chemotaxis/migration) and surveillance (immunoglobulin production/chemokine-related signaling) pathways (Fig. 6B) and decreased expression of signaling and regulation-related gene sets, particularly non-coding RNA-mediated mechanisms (Fig. 6C).

## DISCUSSION

The impact of a short-term AD in HIV(+)MSM and controls was assessed in a controlled clinical trial. High-fat diets can exacerbate SIV-induced immune activation and pathogenesis (52). Conversely, high-fiber, low-fat diets correlate with reduced inflammation and improved metabolic outcomes (57, 89–92). PRBP microbiomes of MSM at risk for HIV infection are compositionally similar to those from agrarian populations (36, 45), and PRBP microbiomes in other contexts have been shown to have either positive or negative associations with metabolic health depending on dietary intake (48, 49, 56–61, 63, 64). Because past studies that conducted high-fiber diet interventions showed greater benefits in individuals with PRBP microbiomes, including greater weight loss in obese individuals (57–59), improved glucose metabolism (60), and a greater improvement in lipid profiles in men with metS (61), we hypothesized that MSM with PRBP microbiomes would have strong health benefits from an AD intervention. Consistent with our hypothesis, we found that high *Prevotella* and low *Bacteroides* at baseline predicted greater reductions in LDL-C with an AD. An opposite trend was observed in the WD, emphasizing that the PRBP microbiome can have different effects depending on diet.

We showed that PRBP microbiomes are less functionally versatile in carbohydrate substrate utilization potential, which is consistent with some past work showing the *Bacteroides* enterotype to have more diversified carbohydrate active enzymes (93) but also surprising, given the prevalence of PRBP microbiomes with plant-based diets and known associations between these diets and enhanced potential for carbohydrate utilization within Prevotellaceae (94). However, starch utilization PULs were enriched in PRBP microbiomes, which is consistent with the ability of starch to sustain the growth of *Segatella* (formally *Prevotella*) *copri* in batch fermenters (93). The AD intervention was rich in resistant starch, a fermentable dietary fiber known to correlate with reduced inflammation and improved cardiometabolic outcomes in humans, including those with metS (95–97). The correlation of LDL-C improvement within individuals with microbiomes preferentially capable of utilizing starch substrates suggests that more refined diet interventions could target increases in resistant starch. Further work is needed to understand the degree to which these patterns in carbohydrate-substrate utilization potential are generalizable across PRBP microbiomes in different contexts. Although PRBP-microbiomes have been shown to have strain-level similarities with non-Western populations (36), functional comparisons of carbohydrate utilization potential have not been made. Although we did include some MSW in our study, the number was small, and they generally had *Bacteroides*-rich/*Prevotella*-poor microbiomes, which made us underpowered for determining whether there were different associations between PRBP microbiomes and diet intervention response in MSM versus MSW. However, including MSW that tended to be more *Bacteroides*-rich allowed us to have better representation of lower *Prevotella* microbiomes in the analysis.

Although we were limited to a 4-week short-term intervention, a stepwise intervention of provided meals followed by guided home preparation allowed for a longer intervention at reduced expense. Although self-feeding resulted in variable compliance, AD macronutrient targets were maintained and reported in sufficient detail for generalizability and future reproducibility. A WD control arm also allowed us to identify effects of the intervention itself, not specific to AD. Expected dietary changes were observed with the AD, whereas subtle changes were detected in the WD compared with baseline. This was an expected finding because although the WD was based on American diets, the baseline diet varied by individual. Meals were prepared using hygienic conditions, limiting exposure to pathogens. This could be a driver of the decreased LBP at T2 in HIV(+)MSM on the WD. HDL-C and LDL-C were responsive to the AD intervention, particularly in HIV(+)MSM. The AD-associated decrease in LDL-C suggests decreased potential incidence of CVD and diabetes (98, 99). Specifically, median reductions in LDL-C of 0.4138 mmoL/L at T2 and 0.2845 mmol/L at T3 have been associated with an 11.6% (T2) and 8.0% (T3) reduced risk of CVD (100). Although this study was limited to a 4-week intervention, these findings suggest potential long-term health benefits of an AD. Although HOMA-IR and triglycerides did not change with the AD, this could be due to the limited representation of individuals with high baseline levels, which was associated with less reduction.

Decreased HDL-C observed with AD may be concerning because HDL-C can have health benefits (101). HDL-C has previously been shown to drop in similar interventions (102–104). In our intervention, reduced HDL-C was not sustained at T3 and did not decrease in participants with low baseline levels. Also, HDL pools can differ in function, being pro- or anti-inflammatory with altered cholesterol efflux capacity; thus, LDL is a more accurate predictor of CVD (105). Furthermore, HIV infection is associated with impaired HDL antioxidant function, complicating its role in PLWH (105). Plasma inflammatory markers did not significantly change with AD. However, when limiting analysis to individuals with higher baseline values, IL-6 was reduced in the AD but not the WD arm. This suggests AD could specifically benefit those with higher baseline inflammation. Although a previous study showed increased inflammatory measures in SIV-infected macaques fed a high-fat diet, it explored untreated infection, where inflammation would be elevated compared with this study population on effective ART (52).

AD decreased blood immune cell populations, including PD-1+ and CD40+ T cells and total monocytes, as well as differential classical and intermediate monocytes. This result trended the same across cohorts and was not related to baseline microbiome composition. Reduced T cells expressing the exhaustion marker PD-1 are specifically relevant to PLWH, since PD-1 expression in T cells is positively associated with HIV replication (106–111), suggesting a potential dietary impact on viral replication. Regarding CD40, a high-cholesterol diet has been shown to increase CD40 immunopositivity in developing atherosclerotic plaques in animal models (112). CD40 can also play a pathogenic role in atherosclerosis onset (113, 114). These findings suggest that AD could confer beneficial metabolic effects by reducing CD40+ T cells. The reported changes in monocytes are not surprising, as fasting reduces circulating monocytes, particularly CD14+ CD16+ intermediate monocytes, and reduced peripheral monocyte number has been linked to dietary protein and glucose (115). Furthermore, because monocytes participate in atherosclerotic lesion progression and CD14+ CD16+ intermediate monocytes are associated with heart disease, these AD-associated changes are clinically relevant (116–118).

No AD-associated biopsy immune cell populations were observed, possibly due to the limited sample number. However, RNA sequencing analysis revealed AD increased expression of host genes related to immune cell trafficking (e.g., neutrophil/granulocyte migration) and surveillance, suggesting potentially increased capability to respond to infection. One potential mechanism for this observation is that short-chain fatty acids (SCFAs), common metabolites of dietary fiber, induce the trafficking of neutrophils to inflamed tissue (119–122). AD also reduced the expression of regulatory genes and altered the expression of non-coding RNAs. Diet, particularly fat intake, has been shown to impact miRNA regulation, with implications for lipid metabolism (123, 124). Furthermore, tRNAs and other non-coding RNAs correlate with altered host lipid metabolism (125, 126).

In this study, MSM differed from MSW by having relatively PRBP gut microbiomes, as has been reported (16, 17, 28–36). Because PRBP gut microbiomes are associated with ADs, it is not surprising that gut microbiomes only had subtle changes with AD intervention, as individuals perhaps already had gut microbiomes suited to an AD (39, 42, 46). The results describing microbiome changes in *Prevotella* and *Bacteroides* from past interventions have been mixed. One study reported that *Prevotella* increased from short-term fiber dietary interventions, whereas another supported the stability of microbial *Prevotella* and *Bacteroides* from short-term changes in fiber and fat (46, 60). However, baseline microbiome characteristics predicting dietary response are consistent with past studies (58–60, 83). One potential explanation for this is that diet is impacting host phenotype through changing the metabolic output of the microbiome. Future studies would benefit from analysis of fecal transcriptomics and metabolomics to assess changes in activity. Although diet only induced subtle changes in the fecal microbiome, we found that MSW had relatively stable microbiomes compared with MSM. Drivers of this heightened variability could include sexual behaviors that increase exposures to different microbiomes or inherent PRBP microbiome attributes. MERF analysis with EXPLANA identified that changes in the relative abundance of specific genera had predictive power for AD-associated changes in LDL-C. The relationship between decreasing levels of *Collinsella* over time and a reduction in LDL-C is consistent with studies associating *Collinsella* with both poor metabolic health and low dietary fiber intake (127). The same relationship with *Parabacteroides* is surprising, given protective relationships previously reported between *Parabacteroides* and metabolic health (128), suggesting that dietary context may be important.

The PRBP microbiomes of MSM have been associated with higher systemic immune activation and induction of gut immune activation in gnotobiotic mice, suggesting negative health effects (38). However, these results suggest a health benefit of this microbiome type in MSM, whereby the altered composition of commensal gut microbes confers a greater capacity of AD-associated metabolic benefit not observed on the WD (58–61). A diverse agrarian-type microbiome in MSM has been linked with sexual behaviors that increase exposure to pathogenic bacteria, including a high number of sexual partners (36). This work suggests that exposure to diverse microbes through sexual behaviors in high-risk MSM may also increase exposure to beneficial commensals.

This study identified beneficial effects of a short-term AD intervention in MSM with PRBP microbiomes, including PLWH. By lowering LDL-C, T-cell exhaustion, and IL-6 in participants with elevated inflammation, AD can reduce metabolic and immune dysfunction in PLWH. Furthermore, the response of PRBP microbiomes to AD and AD-associated host transcriptional changes provides interesting preliminary findings for future work. By demonstrating the clinical utility of dietary matching an AD to PRBP microbiomes, we provide novel insight into potential personalized nutritional strategies to mitigate HIV-associated cardiometabolic complications in MSM.

## MATERIALS AND METHODS

### Enrollment

Recruitment for this study took place at the Clinical Translational Research Center (CTRC) of the University of Colorado Anschutz in Denver, Colorado, between February 2016 and January 2020. Participants were aged 18–65 years with a body mass index between 21 and 29 kg/m^2^ and a stable body weight for at least 3 months. Exclusion criteria included recent systemic antibiotic use, chronic infections (like hepatitis B or C), active malignancy, or diabetes. MSM status and information on sexual behaviors were obtained by questionnaire. HIV(+)MSM had HIV-1 infection defined as a positive antibody test or plasma HIV-1 RNA and were ART-treated (minimum of three drugs) for at least 12 months with no treatment changes or HIV-1 RNA >50 copies/mL within the preceding 6 months. HIV(−) participants had a negative third-generation HIV antibody/antigen screening test. For the optional colonic biopsy, participants with active gastrointestinal disease, a history of bowel resection bleeding, or recent use of high-dose glucocorticoids or alpha-interferon were excluded. Additional details can be found on clinicaltrials.gov (NCT02610374).

### Study design and diet intervention

The experimental control trial was randomized and unblinded. Participants were assigned to a diet using an Excel random number generator. The initial primary outcome was plasma IL-6 to quantify systemic inflammation. Secondary outcomes included fasting triglycerides, HDL-C, LDL-C, HOMA-IR, CRP, and LBP. The target sample size was 50 HIV(+)MSM, 24 HIV(−)MSM, and 24 HIV(−)MSW, determined to detect clinically relevant changes in IL-6 with high statistical power (78, 129). The sample size was calculated using the G*Power software package (130).

Participants completed a 4-week diet intervention. Baseline diets were surveyed using the DHQ2 of the National Cancer Institute (71). Macronutrient totals were estimated using the Diet*Calc Analysis Program version 1.5.0 (131). For the first 2 weeks, food was prepared by the Colorado Clinical and Translational Sciences Institute (CCTSI) Nutrition Core at the University of Colorado Anschutz. Sample menus for each diet are provided in Fig. S9. Total energy intake for each individual was determined using the Mifflin-St. Jeor equation to estimate resting metabolic rate (RMR) with an activity factor of 1.4 to account for daily physical activity (132, 133). Participants completed a survey of food preferences and allergies, which was used to create a personalized 4-day menu that was repeated 3.5 times. Participants were instructed to eat only the provided food, and uneaten or extra food was recorded. For the final 2 weeks, participants prepared their own meals with guidance from the CCTSI Nutrition Core. A 24-h food recall was completed weekly and analyzed using the Nutrition Data System for Research software. Adherence was quantified using the Euclidean distance from the target macronutrient totals at T2 and T3 from—T2, provided diets adjusted by macronutrient totals of participant reports of uneaten and extra food. T3, application of Nutrition Data System for Research software to 24 h food recall data.

### Sample collection

Fecal samples were collected at T1, T2, and T3 using a commode specimen collector and stored at −80°C. Whole blood was collected in sodium heparin vacutainers and centrifuged at 1,700  rpm for 10 min for plasma collection. Plasma was aliquoted into 1 mL microcentrifuge tubes and stored at –80°C. Participants were given two Fleet saline enemas (Prestige Consumer Healthcare, Inc., Lynchburg, VA, USA), followed by flexible sigmoidoscopy with collection of 30 pinch colorectal biopsies using 2.4 mm forceps. Two-to-four pinch biopsies were put into PBS for microbiome analysis, and, in a subset of participants, two pinch biopsies were put in 0.25 mL of RNALater (Thermo Fisher Scientific, Waltham, MA, USA) for transcriptomics, both stored at −80°C. The remaining biopsies were put in 10 mL low-barium PBS for use with CyTOF. The biopsies were digested for 1.5 h with collagenase (1 mg/mL) and DNAse (5 μL/mL) as previously described (134). Pinches were then mashed on and filtered through a 70 μm nylon cell strainer and washed with 15 mL low barium PBS, centrifuged, and resuspended in 2 mL low barium PBS. Cells were divided and immediately stained for CyTOF.

### Immune and metabolic data collection

For CyTOF, fresh plasma and biopsy single-cell suspensions were stained for live–dead cell distinction using 2.5 µM cisplatin (Fluidigm, South San Francisco, CA, USA). Cells were re-suspended in 65 µL barium-free FACS buffer (low barium PBS with 0.1% BSA and 2  mM EDTA) and incubated for 30 min at 4°C with a 35 µL cocktail of metal-conjugated antibodies (1 µL each) (Table S1). Cells were washed and resuspended with MaxPar fix with DNA intercalator (0.125 µM Iridium-191/193; Fluidigm, South San Francisco, CA, USA), and EQ Four Element Calibration Beads (Fluidigm, South San Francisco, CA, USA) were added. Cells were acquired using a CyTOF2 mass cytometer (Fluidigm, South San Francisco, CA, USA). CyTOF software v.6.0.626 was used with noise reduction, a lower convolution threshold of 200, event length limits of 10–150 pushes, a sigma value of 3, and a flow rate of 0.045  mL/min.

Plasma inflammatory markers, LBP, CRP, and IL-6, were measured using the following enzyme-linked immunosorbent assays (ELISAs): Hycult Biotech Kit HK315-02, Millipore Sigma–PAB0096-1KT, and Invitrogen-88-7066.22, respectively. For ELISA preparation, plasma was thawed, kept cold, and centrifuged at 2,000  ×  *g* for 20 min before plating, and the manufacturer’s protocols were followed. In brief, plasma and standards were diluted per manufacturer’s protocol in sample diluent and added to pre-coated microplate wells. Following incubation of the wells with biotinylated detection antibody, HRP conjugate, substrate reagent, and stop solution was added, and the plates were read at 450 nm using a microplate reader with Softmax Pro Software from Molecular Devices LLC. Metabolic markers (HDL-C, LDL-C, triglycerides, glucose, and insulin) were measured at the CTRC at the University of Colorado Anschutz, and HOMA-IR was calculated (135). Healthy thresholds for lipid concentrations and HOMA-IR were defined based on previously published data (75–77).

### Sequencing and analysis

DNA was extracted from fecal and biopsy samples as well as batch blanks using a Qiagen DNeasy PowerSoil Kit for both 16S rRNA and shotgun metagenomic gene sequencing. To monitor for potential contamination, a blank was included in each extraction batch and processed using the same extraction protocol as the samples. DNA concentrations of extraction blanks were measured, and blanks were carried through PCR amplification. PCR products were quantified, and any blank with a PCR product concentration exceeding 12 ng/µL was pooled and sequenced alongside samples. In addition, multiple PCR blanks (2–4 per library) were included in each sequencing library, prepared using water as the template. PCR product concentrations for these blanks were similarly quantified, and any exceeding 12 ng/µL were also pooled and sequenced. 16S rRNA gene sequencing targeted the V4 region with the 515F:806R primer constructs in accordance with Earth Microbiome Project protocols (136). PCR products were quantified with Picogreen (Invitrogen), and equal amounts of DNA were pooled, followed by cleaning with UltraClean PCR Clean-up Kit (MoBio). 16S sequencing data were obtained from a MiSeq personal sequencer using the V2 chemistry 500-cycle cartridge (Illumina, San Diego, CA). Microbiome 16S rRNA analysis was conducted using QIIME 2 version 2022.8.0 (81), with raw sequences demultiplexed and denoised using the DADA2 plugin (137). Amplicon sequence variants (ASVs) were taxonomically classified using the RDP classifier as implemented in QIIME 2 and the Greengenes2 database (138). Taxa classified as chloroplasts or mitochondria were excluded. SATé-Enabled Phylogenetic placement (SEPP) was used to generate a phylogenetic tree (139). The tree was then used for measuring Faith’s Phylogenetic Diversity (140) and weighted UniFrac distances (141). Samples were rarefied to 2,790 and 12,922 reads per sample for biopsy and feces, respectively.

Shotgun sequencing was also performed on DNA from samples collected as a baseline. The gDNA purity, quantity, and size distribution were determined with Qubit (Invitrogen) and TapeStation 4200 (Agilent) analyses prior to DNA-seq library preparation. An input of 55 ng of the gDNA was mechanically sheared (Covaris), targeting 300–400 bp DNA products, and the Ovation Ultralow System V2 kit (Tecan) was used to generate DNA-Seq libraries. Paired-end sequencing reads of 150 bp were generated on the NovaSeq X plus (Illumina) sequencer at a target depth of 600 million paired-end reads per sample. Raw sequencing reads were de-multiplexed using bcl2fastq. Raw reads were trimmed with trimmomatic (142) to remove ILLUMINACLIP adapters, allowing two seed mismatches, using a palindrome clip threshold of 30 and a simple clip threshold of 10, and to perform quality trimming with a 4-base sliding window, a minimum end quality of 20, and a minimum read length after trimming of 50. As is standard for Illumina sequencing, base quality was assessed using the Phred + 33 scale (143), quality checked with FASTQC and MultiQC, and hostile host-filtered with the masked human-t2t-hla-argos985 human reference genome using bowtie2 alignment (142, 144–146). Contig assembly was performed using Megahit with a minimum contig length of 1000 base pairs (147), and annotation was done with Prokka (148). Carbohydrate-active enzymes and substrate targets were quantified using dbCAN3 and SAMtools (87, 149).

RNA was extracted from the biopsy for metatranscriptomics using a Qiagen AllPrep kit according to the manufacturer’s protocol with modifications to support the isolation of high-quality human and microbial nucleic acids. Specifically, the sample was bead-beaten for 7 min, and lysis was continued for an extra 40 min with rotation end over end. The sample was then frozen at −80°C overnight, thawed in a 37°C water bath, and rotated in lysis buffer for an additional hour before conducting the remainder of the manufacturer’s protocol. Quality control was performed using a ThermoFisher NanoDrop 2000 spectrophotometer, which measured both concentration and 260/280 nm ratio. Ribo-depleted libraries were constructed by the University of Colorado Genomics core using the Zymo-Seq RiboFree Total RNA Library Kit (Zymo Research). Libraries underwent quality control via tape station prior to multiplexing. Sequencing was performed by the University of Colorado Genomics core on an Illumina NovaSeq6000 (San Diego, CA, USA) to a depth of 100 M reads per sample. Raw reads were trimmed and quality checked using the same tools and parameters used for shotgun sequencing reads, but were aligned to the GRCh38 human reference genome using HISAT2 and quantified with featureCounts (150, 151). Functional predictions were determined using Human Microbiome Project (HMP) Unified Metabolic Analysis Network (HUMAnN) from bioBakery3 (152).

### Statistical analyses

Cross-sectional comparisons were performed using Wilcoxon rank-based and Kruskal-Wallis tests, with Dunn’s *post hoc* tests for pairwise comparisons. For microbiome data, ANCOM-BC was used for pairwise and longitudinal comparisons, stratifying by cohort and by diet, incorporating HIV and MSM status as confounders and study ID as a random effect (80). Longitudinal changes in other markers were analyzed using Friedman tests and linear mixed-effects models (LMEMs), stratifying by diet, incorporating HIV and MSM status as confounders, and study ID as a random effect. The false discovery rate (FDR) correction of Benjamini and Hochberg was applied to adjust for multiple comparisons (153). Adonis statistical tests were used to assess differences in overall microbiome composition (154). Spearman correlations were used to compare changes in LDL-C to baseline genus abundance, using center-log ratio (CLR)-transformed counts to account for compositionality, where one count was added to each field to account for zeros. For RNA sequencing, Limma was used to identify differences (88), and GSEA was performed with the clusterProfiler R package (155). For exploratory analysis, EXPLANA was used to identify features related to LDL-C, and BorutaSHAP was used to determine the importance of features (85, 156). Throughout our analysis, a *P*-value under 0.05 was defined as statistically significant.

## Data Availability

The code and processed de-identified data used for this analysis are available on GitHub (https://github.com/jboconnor13/HIV-Diet-Intervention). The sequence data from the 16S rRNA sequencing and shotgun metagenomic sequencing are both available on ENA (project PRJEB81676, accession ERP165477, and project PRJEB97219, accession ERP179815, respectively).

## SUPPLEMENTAL MATERIAL

Supplemental material msystems.01185-25-s0001.docx Supplemental figures, Table S1, and captions for Tables S2 to S7.

Table S2 msystems.01185-25-s0002.csv Baseline fecal microbiome differences.

Table S3 msystems.01185-25-s0003.csv Fecal agrarian microbiome changes stratified by cohort.

Table S4 msystems.01185-25-s0004.csv Fecal agrarian microbiome changes.

Table S5 msystems.01185-25-s0005.csv Baseline biopsy microbiome differences.

Table S6 msystems.01185-25-s0006.csv Biopsy agrarian microbiome changes stratified by cohort.

Table S7 msystems.01185-25-s0007.csv Biopsy agrarian microbiome changes.

ASM does not own the copyrights to Supplemental Material that may be linked to, or accessed through, an article. The authors have granted ASM a non-exclusive, world-wide license to publish the Supplemental Material files. Please contact the corresponding author directly for reuse.
