# Supplementary material for: Agrarian diet improves metabolic health in HIV-positive men with Prevotella-rich microbiomes: results from a randomized trial
Source: mSystems. 2025 Nov 26;10(12):e01185-25. doi: 10.1128/msystems.01185-25 (PMC12710365; doi:10.1128/msystems.01185-25)
Supplement: Supplemental material — Supplemental figures, Table S1, and captions for Tables S2 to S7. [file msystems.01185-25-s0001.docx]

**Supplement:**


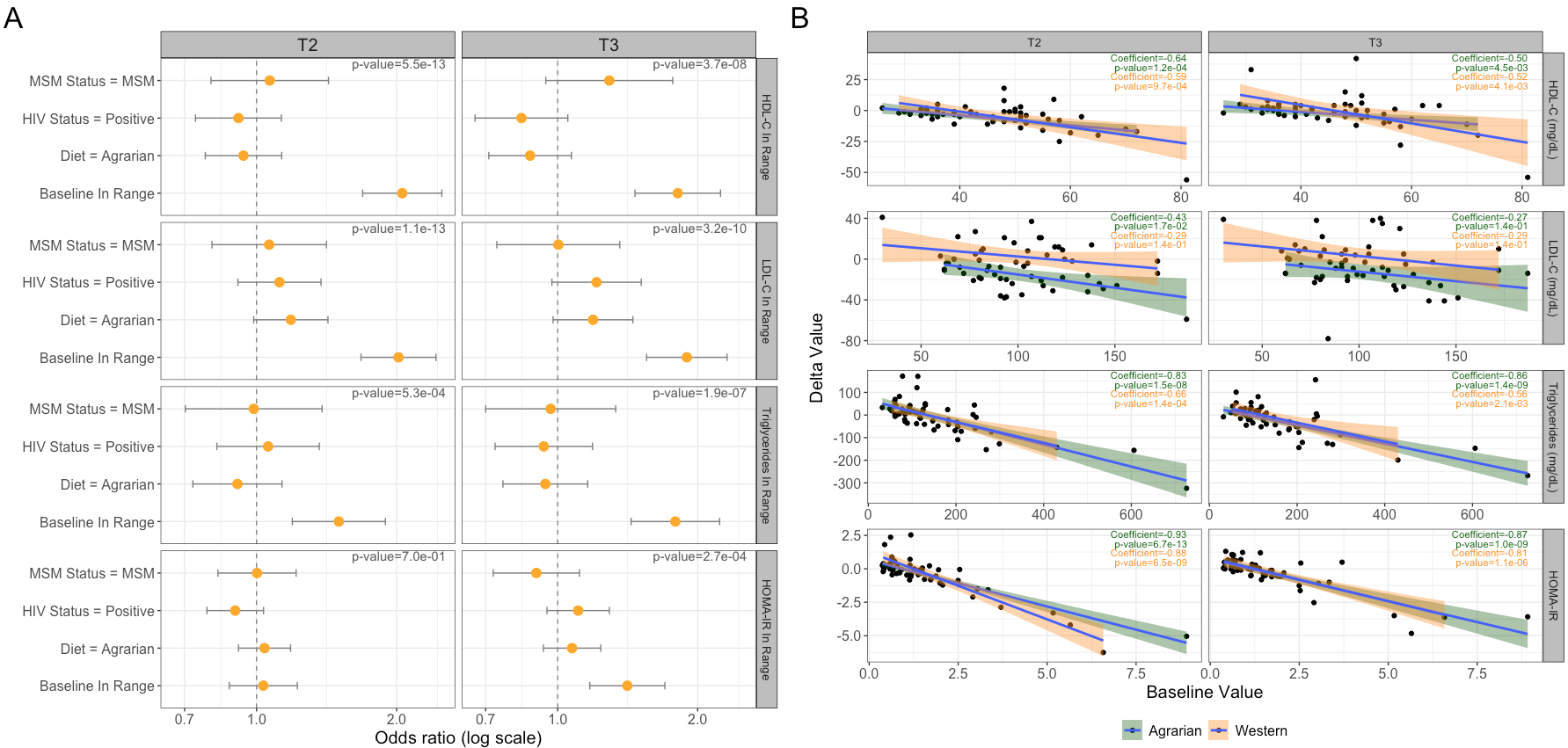


**Figure S1: Baseline Metabolic Measures Predictive of Measures at Later Timepoints.** (A) Results of logistic regressions of metabolic measures being in the healthy range of metabolic markers (row panels) at different timepoints (column panels), y-axis represents predictive variables, x-axis represents odds ratios, brackets indicate 95% confidence intervals, and intercept p-values are in the top right corners; (B) Scatter plots of baseline values of different metabolic measures (row panels) to changes in those values at different timepoints (column panels), x-axis represents baseline values, y-axis represents the change from T1 to T2 (left) and T1 to T3 (right), shading indicates 95% confidence intervals; Spearman correlation coefficients and p-values are displayed in the top right corner. MSM=men who have sex with men, HIV=human immunodeficiency virus, HDL-C=high-density lipoprotein cholesterol, LDL-C=low-density lipoprotein cholesterol, HOMA-IR=Homeostatic Model Assessment for Insulin Resistance.


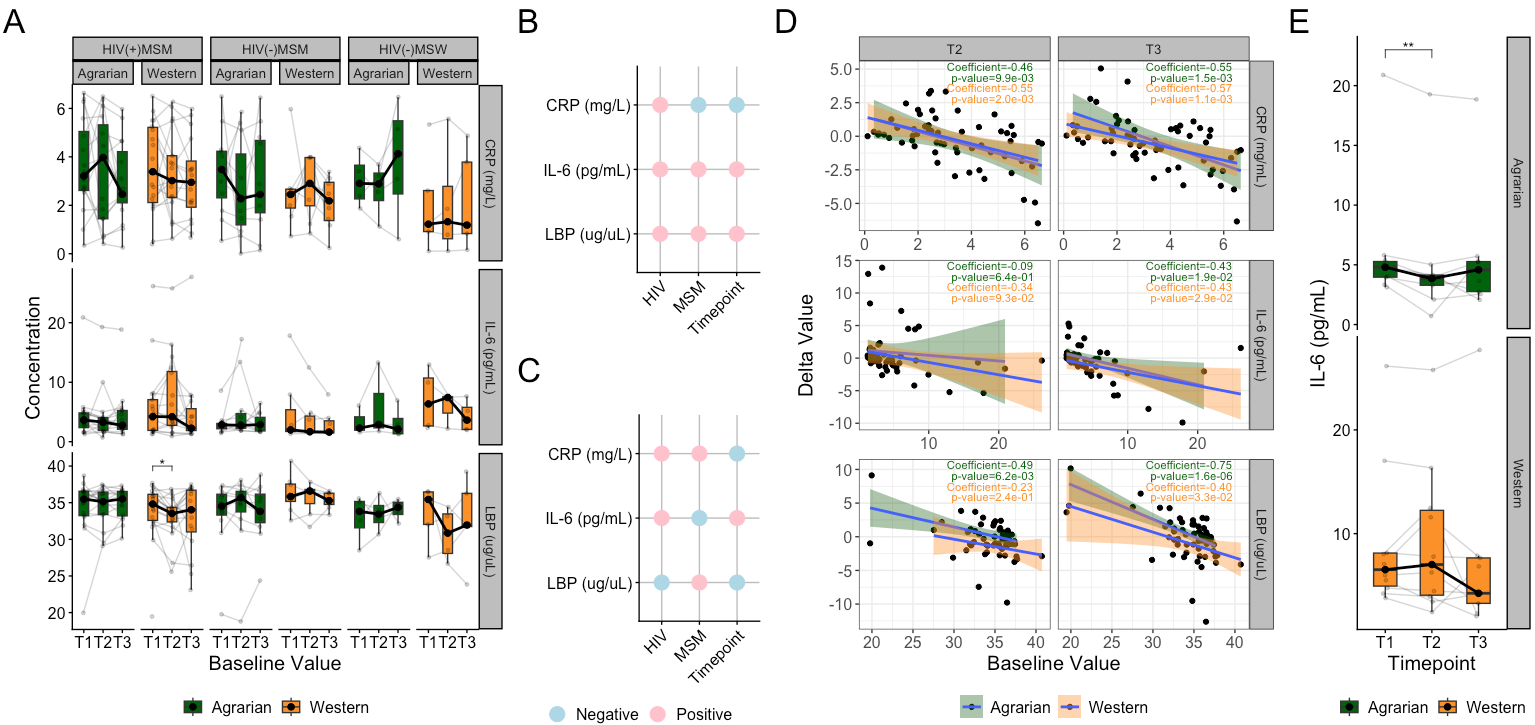


**Figure S2: Baseline Values of Inflammatory Markers and LBP Predictive of Measures at Later Timepoints.** (A) Box plots overlaid with spaghetti plots of inflammatory markers colored by diet with brackets indicating significance as determined by Friedman test with Bonferroni multiple comparisons correction, thin lines and dots represent values from individual participants while thick black lines and dots represent median values ; * p≤0.05; (B and C) Coefficients of linear mixed-effects models (LMEMs) relating inflammatory markers to MSM status, HIV infection status, and timepoint (continuous variable representing time on the diet intervention) in (B) those on the agrarian diet and (C) those on the western diet. Red indicates a non-significant positive relationship while blue indicates the opposite. P-values were determined by analysis of variance (ANOVA) of full LMEM model (Immune Cell Population ~ HIV+MSM+(1|StudyID) vs model removing predictor of interest. (D) Scatter plots of baseline values of different immune measures (row panels) to changes in those values at different timepoints (column panels), x-axis represents baseline values, y-axis represents the change from T1 to T2 (left) and T1 to T3 (right); shading indicates 95% confidence intervals. Spearman correlation coefficients and p-values are displayed in the top right corner. (E) Box plots overlaid with spaghetti plots of IL6 in participants starting with values in the top 50^th^ percentile. Plots are colored by diet with brackets indicating significance as determined by Friedman test with Bonferroni multiple comparisons correction. Thin lines and dots represent values from individual participants while thick black lines and dots represent median values; ** p≤0.01. Brackets indicate significance as determined by Friedman tests with Bonferroni multiple comparisons correction; ** p≤0.01, HIV=human immunodeficiency virus, MSM=men who have sex with men, MSW=men who have sex with women, CRP=C-reactive protein, IL-6=interleukin 6, LBP=lipopolysaccharide binding protein.


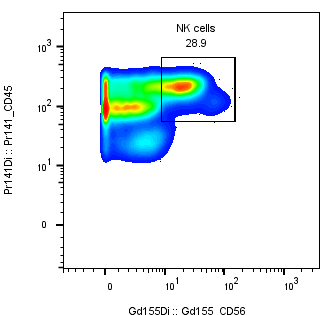

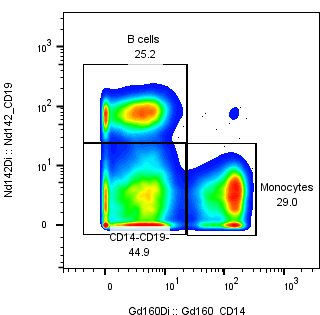

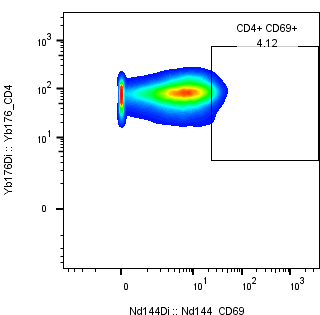

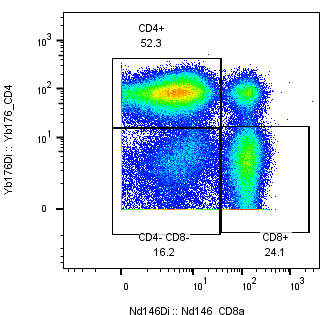

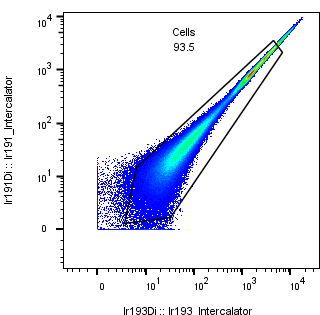

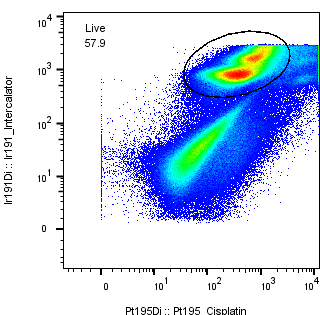

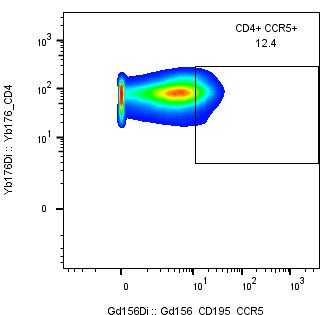

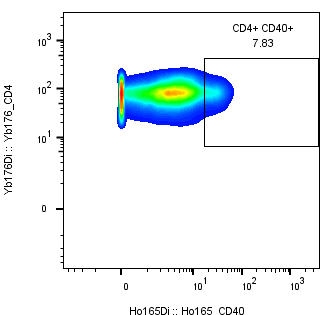

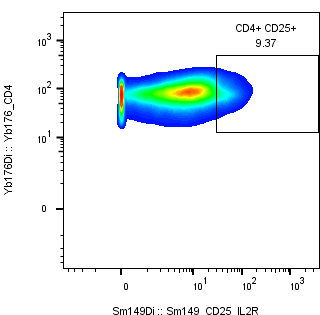

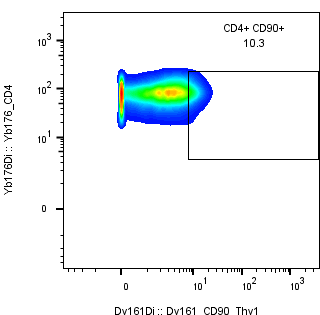

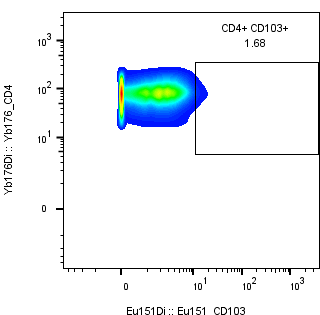

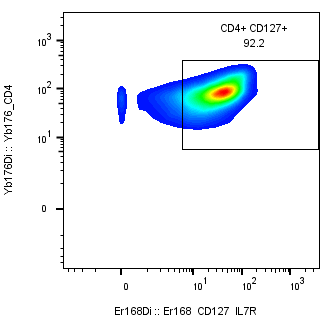

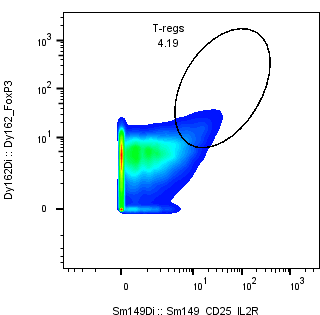

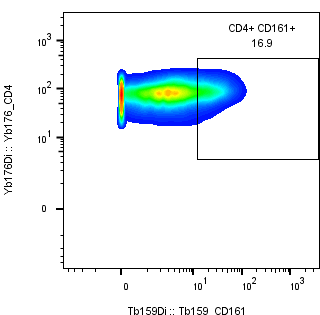

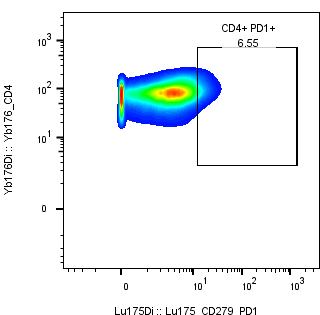

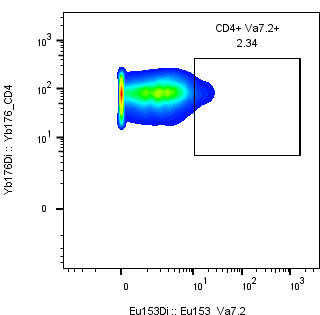

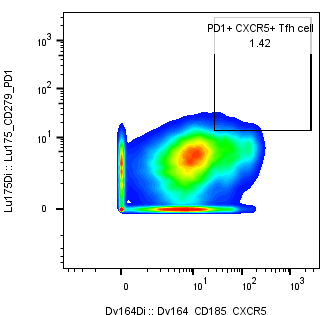

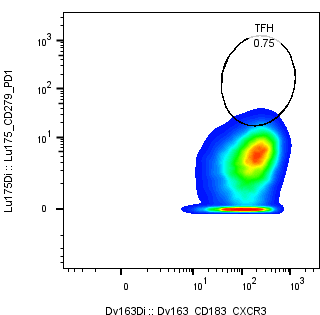

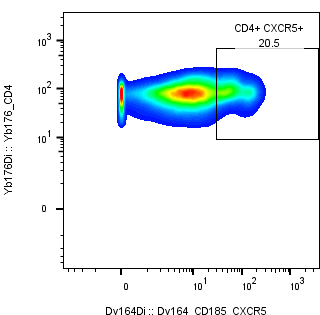

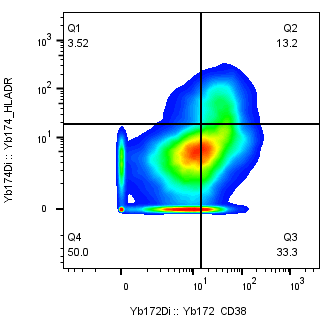

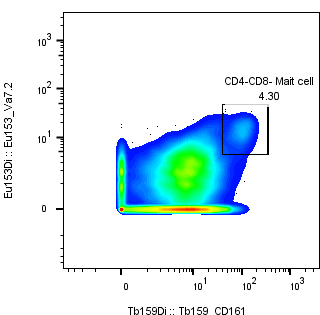

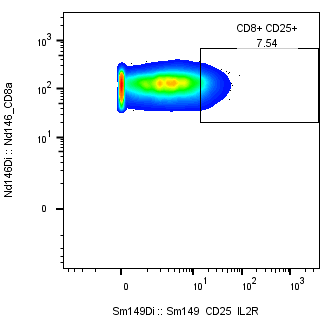

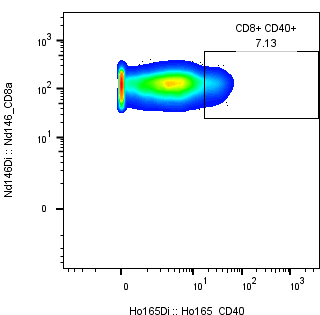

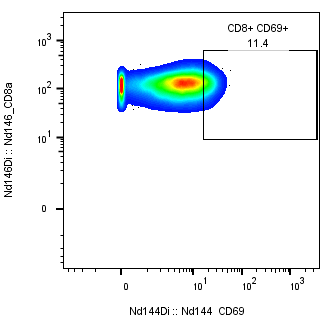

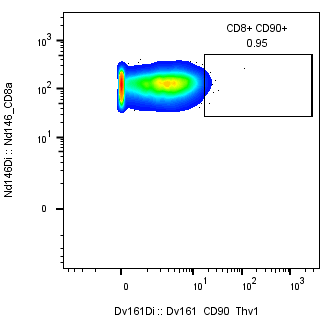

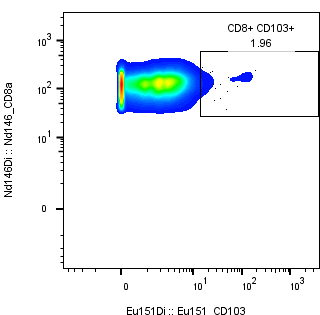

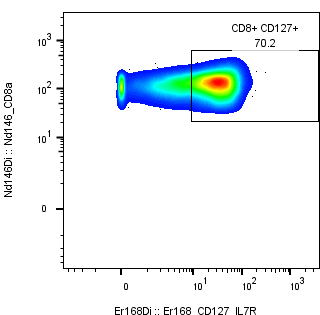

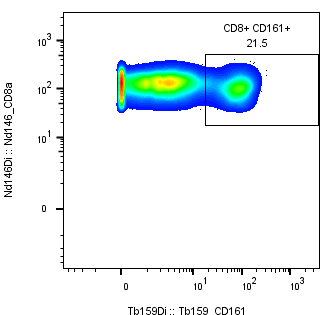

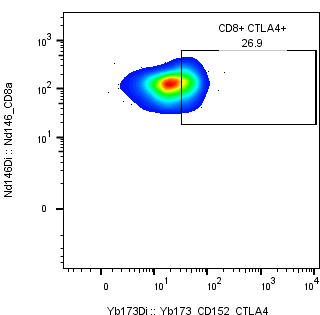

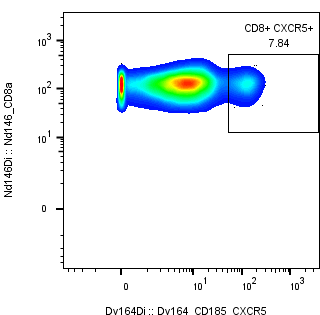

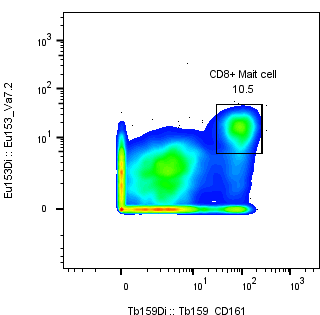

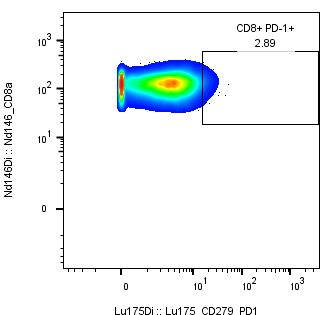

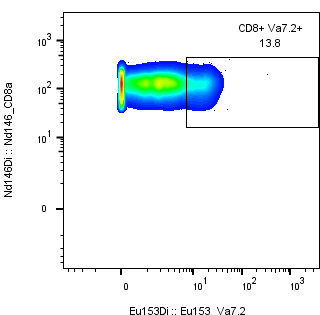

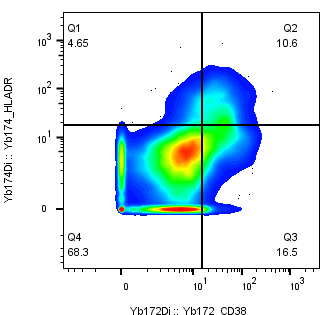

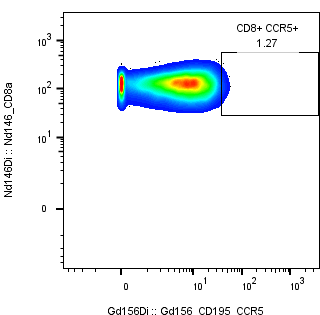

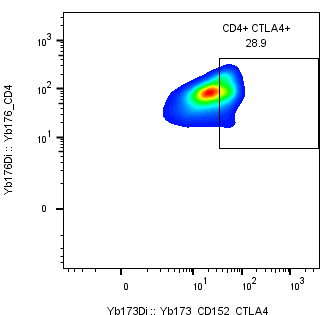

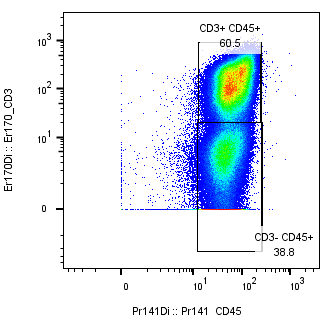

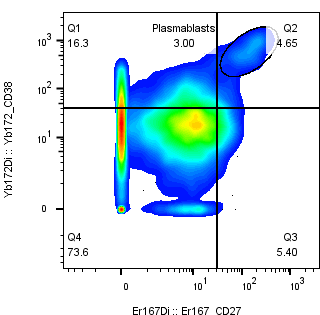

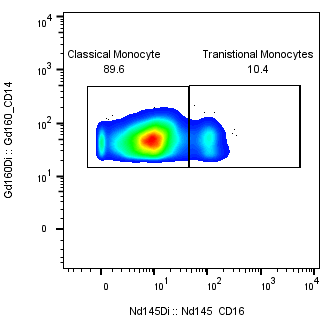

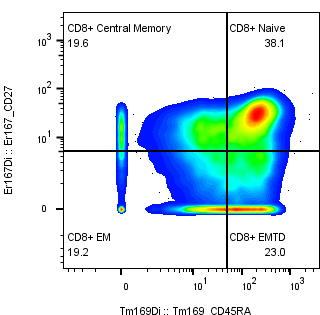

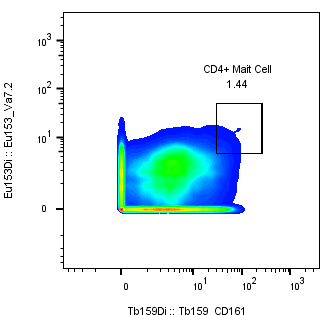

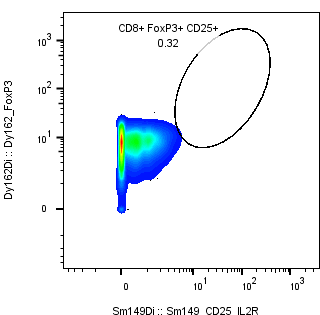

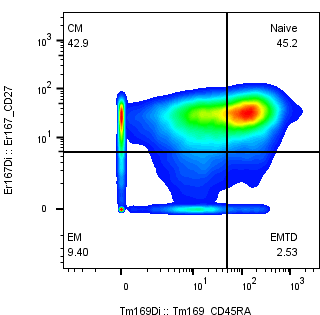


CD4+

CD8+

CD4-CD8-

**Figure S3: Representative CyTOF Gating Hierarchy.** Visual representation of cytometry by time-of-flight (CyTOF) gating strategy from a peripheral blood mononuclear cell (PBMC) sample showing the identification of key immune cell populations. Intact viable cells were selected followed by manual gating of major immune cell subsets including CD4+ T cells, CD8+ T cells, monocytes and B cells, with T cell populations being further evaluated for additional subsets.


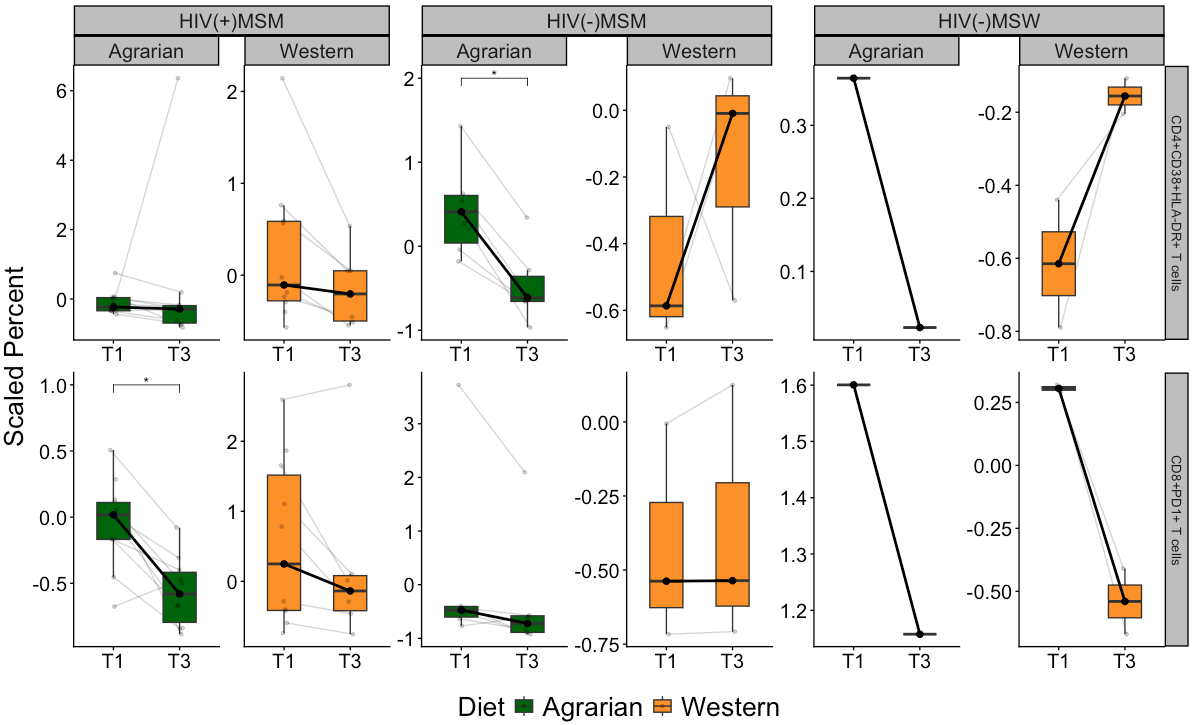


**Figure S4: T Cell Changes Observed in HIV(+)MSM and HIV(-)MSM on an AD.** Box plots overlaid with spaghetti plots of CD8+ PD1+and CD4+CD38+HLA-DR+ T Cells colored by diet with brackets indicating significance as determined by Friedman tests with Bonferroni multiple comparisons correction; *-p≤0.05.


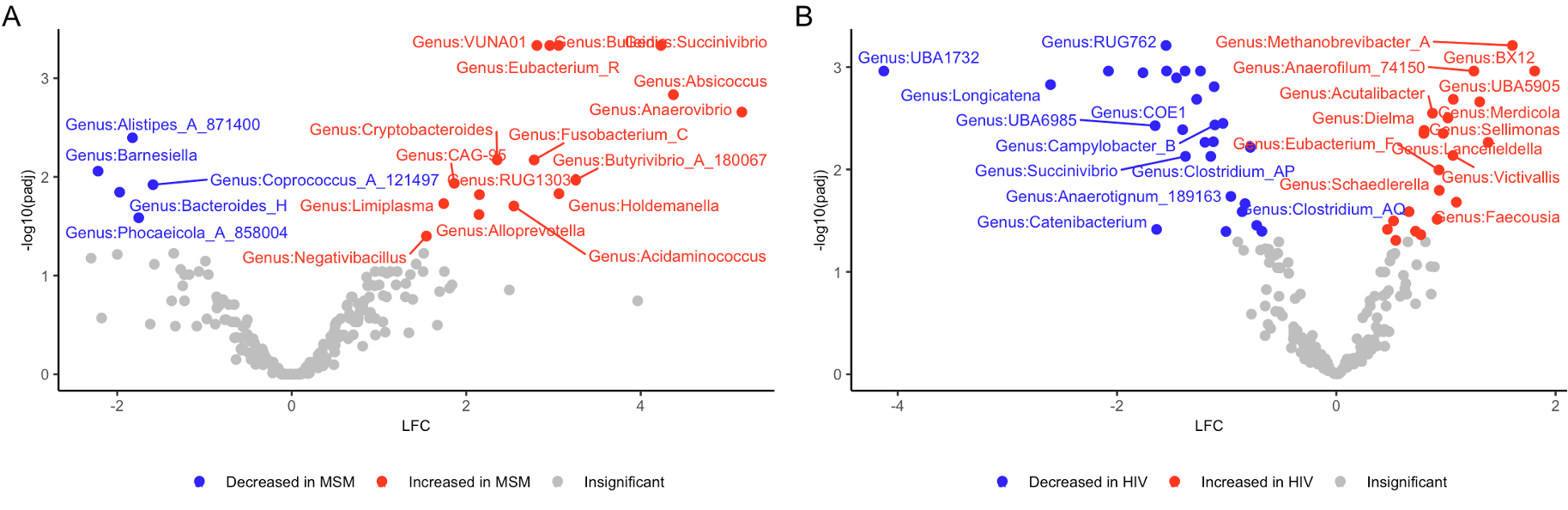


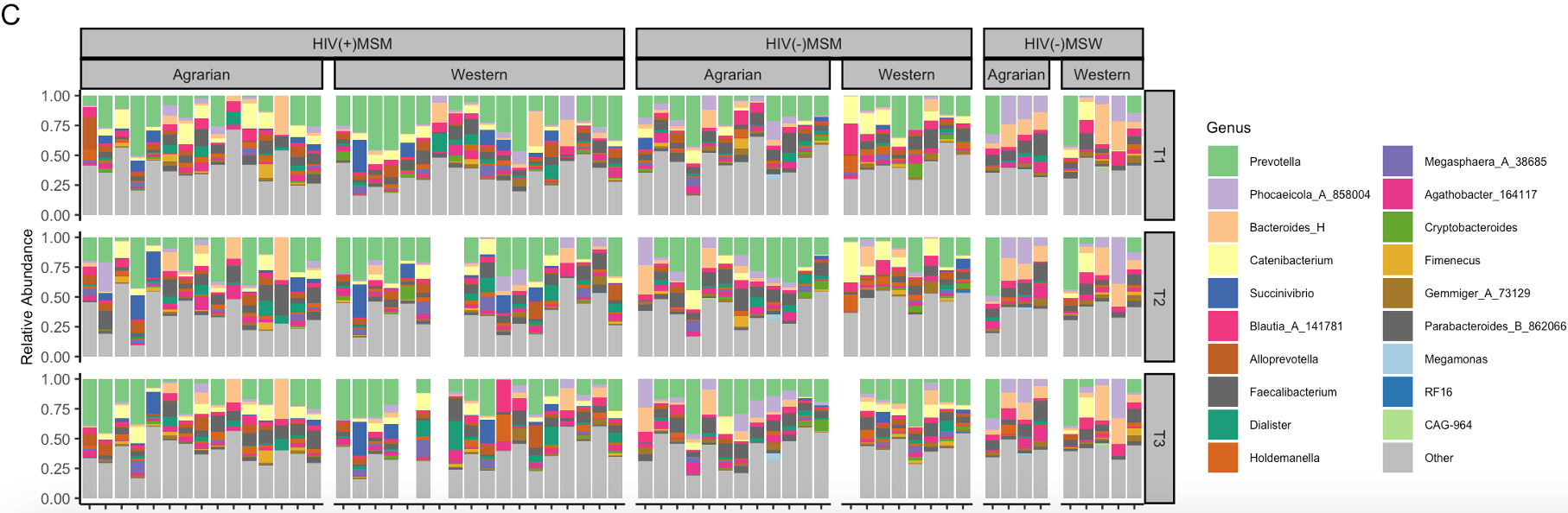


**Figure S5: Fecal Microbiomes Differ by HIV and MSM Status.** Analysis of Compositions of Microbiomes with Bias Correction (ANCOM-BC) results comparing fecal microbiome genera at baseline between (A) HIV(-)MSM and HIV(-)MSW and (B) HIV(+) and HIV(-)MSM; (C) Bar plots of most abundant genera stratified by group, diet, and timepoint; LFC=log-fold change, HIV=human immunodeficiency virus, MSM=men who have sex with men, MSW=men who have sex with women.


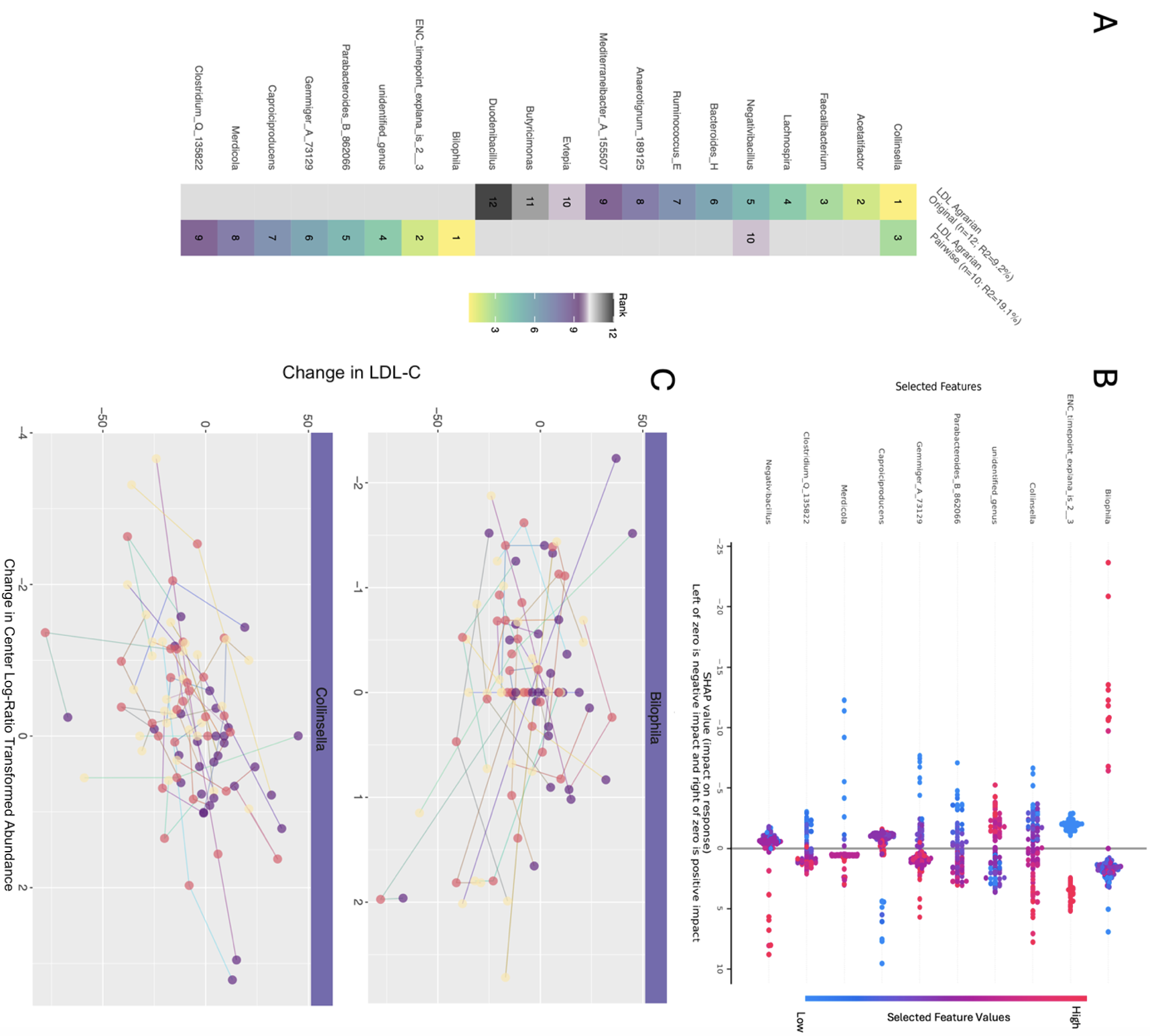


**Figure S6. Features Related to LDL-C in Individuals on an Agrarian Diet Intervention, Selected Using Mixed-effects Random Forests.** EXPLANA (EXPLoratory ANAlysis) software was used to find important features over three timepoints using original values and pairwise changes/deltas in features between timepoint per individual. A) Features selected using original data or pairwise comparisons are ranked, where one is most important. The top ten features per model are emphasized using a sequential, multi-hue color palette from light to dark, and features after ten are grayscale from light to dark. *Collinsella* and *Negativibacillus* were selected by both models, while most genera were unique to original or pairwise. B) SHAP beeswarm plot of top ten features by magnitude of impact on LDL-C using model built from pairwise deltas. Each point represents one sample, and the horizontal position indicates the impact on the outcome as indicated on the x-axis. Points to the left indicate a negative impact and points to the right indicate a positive impact. The colors represent feature values, where red is larger, and blue is smaller. For binary encoded categorical features (‘ENC’) such as timepoint, red is yes/1 and blue is no/0. C) Scatterplot showing pairwise differences in center log-ratio transformed *Bilophila* and *Collinsella* values (top two important genera selected using pairwise comparisons) with respect to LDL-C. Points are colored by timepoint 1_2 (yellow), 1_3 (red), and 2_3 (purple) and lines connect data per individual. Linear mixed-effects models using pairwise deltas confirm LDL-C has a negative relationship to *Bilophila* and a positive relationship to *Collinsella*. LDL-C=low density lipoprotein cholesterol.


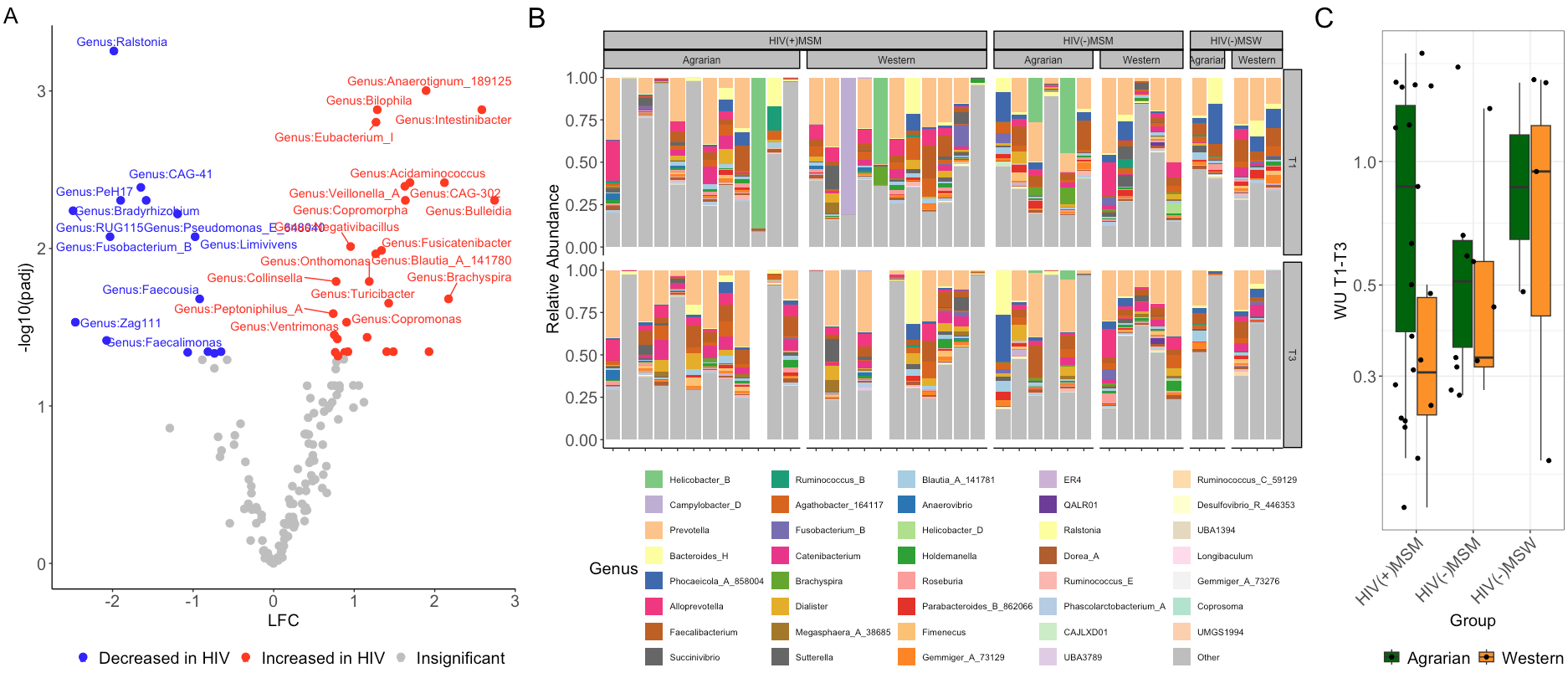


**Figure S7: Biopsy Microbiomes Differ by HIV.** ANCOM-BC results comparing biopsy microbiome genera at baseline between (A) HIV(+) and HIV(-)MSM; (B) Bar plots of most abundant genera stratified by group, diet, and timepoint; (C) Weighted UniFrac distances from baseline to T3. LFC=log-fold change, HIV=human immunodeficiency virus, MSM=men who have sex with men, MSW=men who have sex with women.


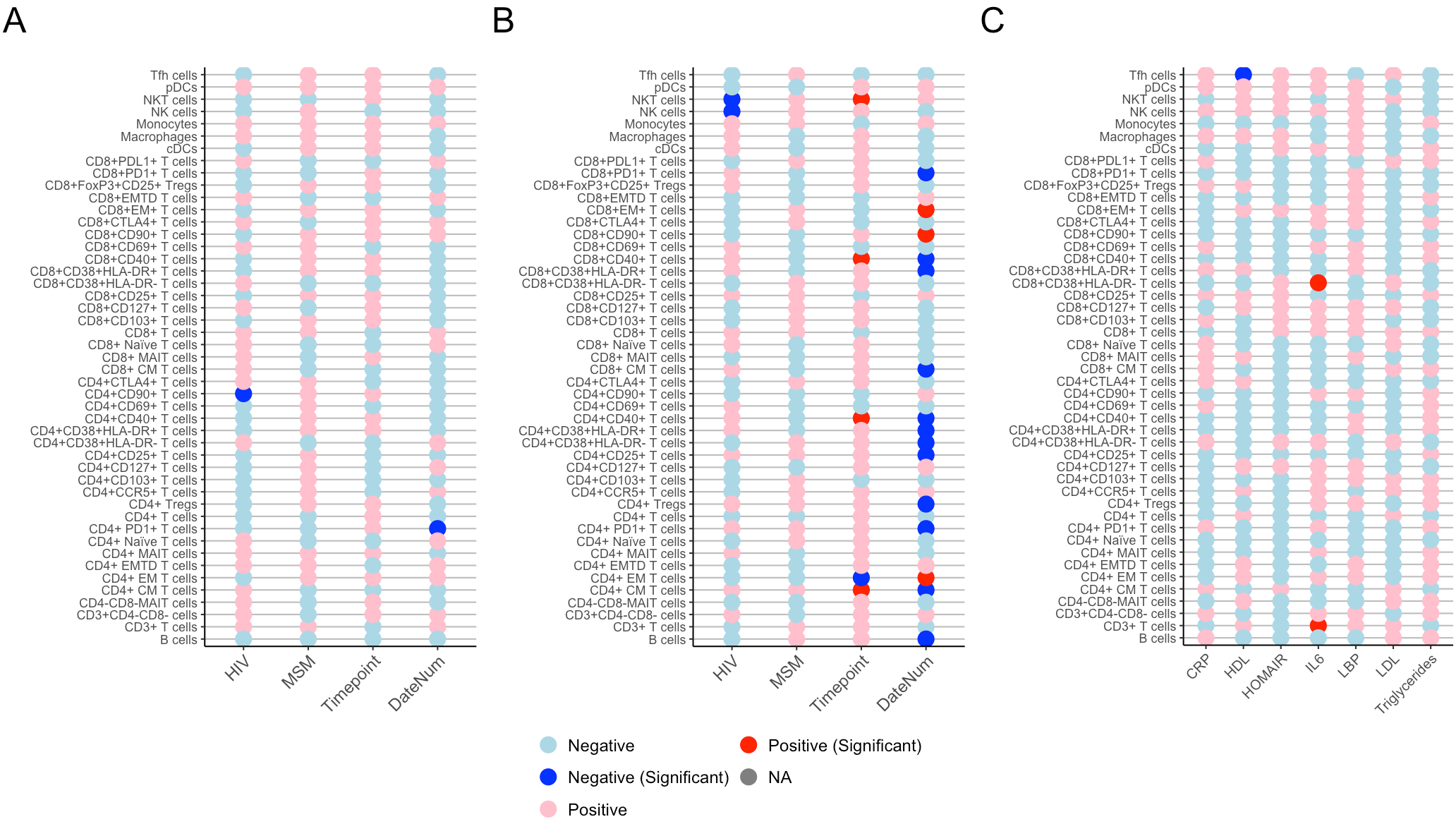


**Figure S8: AD Associated with No Changes in Biopsy Immune Cell Populations.** Coefficients of linear mixed-effects models (LMEMs) relating biopsy immune cells to MSM status, HIV status, and timepoint (continuous variable representing time on the diet intervention) in (A) those on the AD and (B) those on the WD. Red indicates a positive relationship with while blue indicates the opposite. P-values determined by analysis of variance (ANOVA) of full model Cell Population ~ HIV+MSM+(1|StudyID) vs model removing predictor of interest. Significance determined by a p-value under 0.05. (C) coefficients of LMEMs comparing each cell population to inflammatory and metabolic markers. P-values determined by ANOVA of full model (Cell Population ~ CRP+HDL+HOMAIR+IL-6+LBP+LDL+Triglycerides+HIV+MSM+Diet(1|StudyID) vs model removing predictor of interest. Diet, MSM, and HIV coefficients not displayed. LDL-C=low-density lipoprotein cholesterol, HDL-C=high-density lipoprotein cholesterol, LBP=lipopolysaccharide binding protein, HOMA-IR=Homeostatic Model Assessment for Insulin Resistance, IL-6=Interleukin-6, CRP=C reactive protein, DateNum=Continuous variable representing date of the run


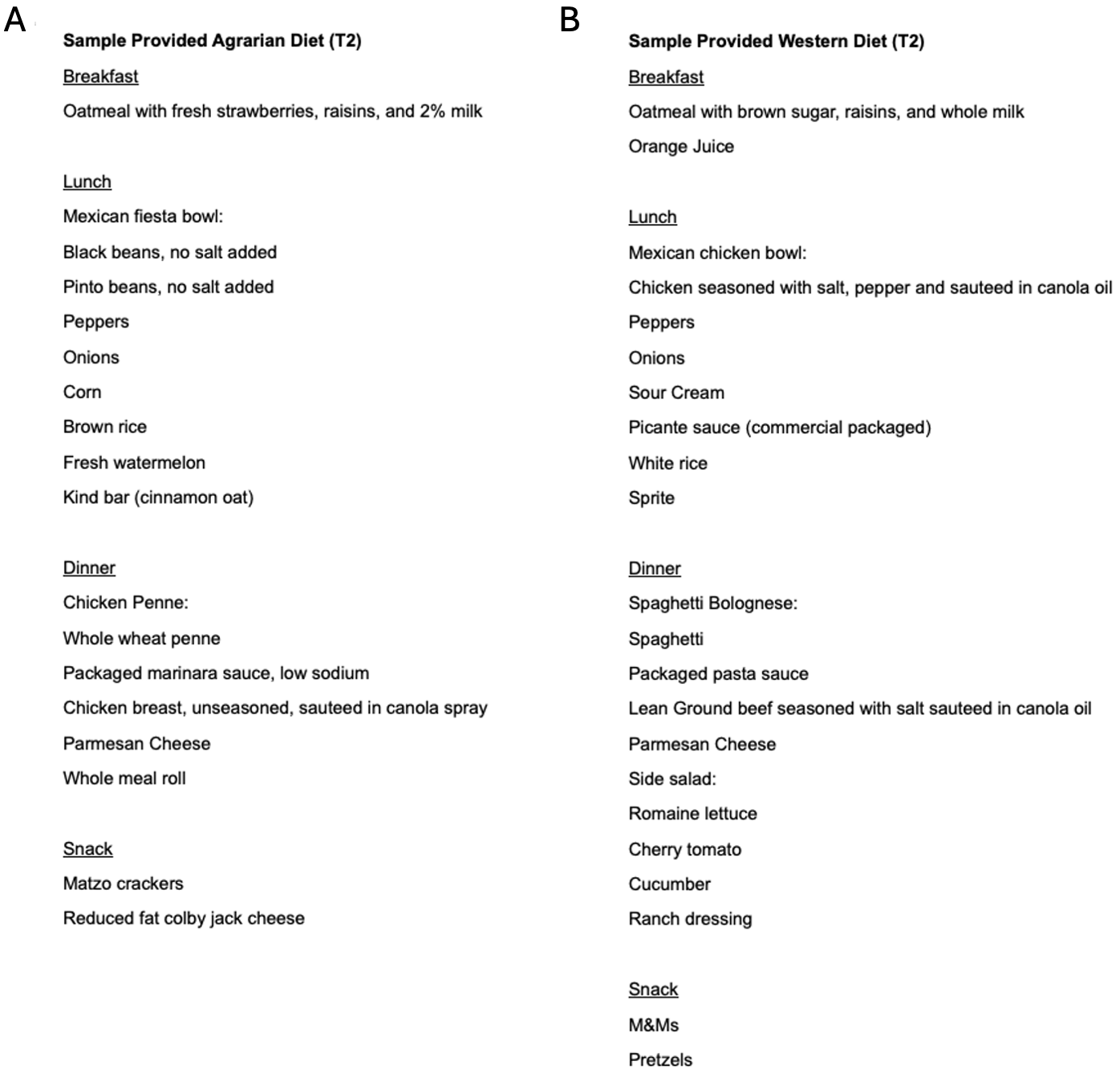


**Figure S9: Sample Menus.** Sample menus for provided meals prepared by the Colorado Clinical and Translational Sciences Institute (CCTSI) Nutrition Core at the University of Colorado, Anschutz Medical Campus for the (A) agrarian and (B) western diet groups.


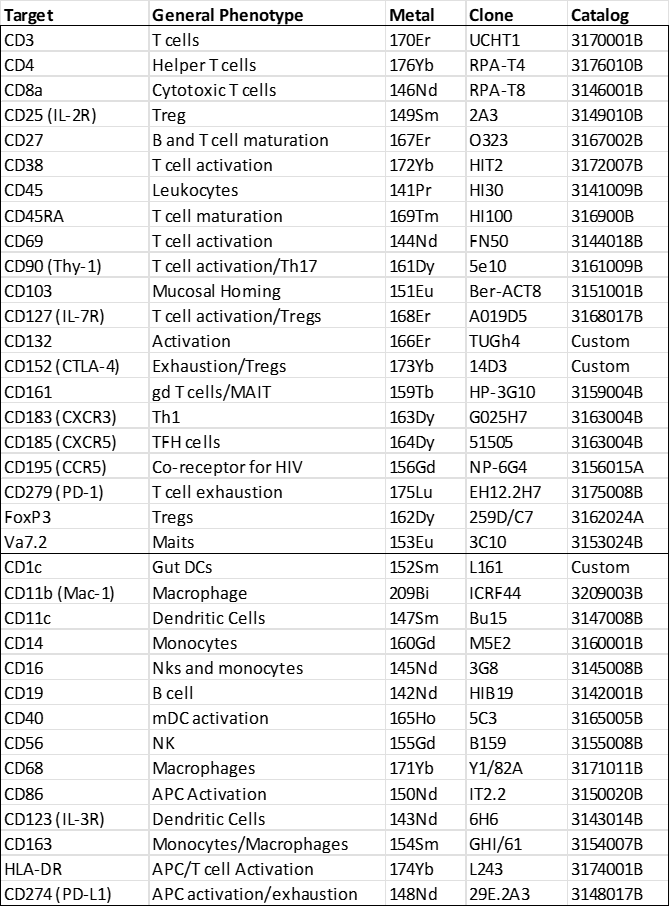


**Table S1:** **CyTOF mAb Panel**: Target antibody panel used to characterize immune populations in blood and colonic biopsy in Cytometry by Time-of-flight mass spectrometry (CyTOF). Treg=regulatory T cell, gd=gamma-delta, MAIT=mucosal-associated invariant T, HIV=human immunodeficiency virus, DC=dendritic cell, mDC=myeloid dendritic cell, NK=natural killer cell, APC=antigen presenting cell.

**Table S2: Baseline Fecal Microbiome Differences.** Results of ANCOM-BC comparing differences in baseline fecal microbiome genus abundance by MSM status (comparing HIV(-)MSM and HIV(-)MSW) and HIV status (comparing HIV(+)MSM and HIV(-)MSM), rows correspond to individual genera and columns include log fold changes (“LFC”), p-values (“p”), false discovery rate adjusted p-values (“q”); LFCs correspond to change associated with HIV and MSM status

**Table S3: Fecal Agrarian Microbiome Changes Stratified by Cohort.** Results of ANCOM-BC comparing fecal microbiome genus abundance to time on the agrarian diet stratified by cohort, rows correspond to individual genera and columns include log fold changes (“LFC”), p-values (“p”), false discovery rate adjusted p-values (“q”); LFCs correspond to change at later timepoints

**Table S4: Fecal Agrarian Microbiome Changes.** Results of ANCOM-BC comparing fecal microbiome genus abundance to time on the agrarian diet accounting for HIV and MSM status in the model, rows correspond to individual genera and columns include log fold changes (“LFC”), p-values (“p”), false discovery rate adjusted p-values (“q”); LFCs correspond to change at later timepoints

**Table S5: Baseline Biopsy Microbiome Differences:** Results of ANCOM-BC comparing differences in baseline biopsy microbiome genus abundance by MSM status (comparing HIV(-)MSM and HIV(-)MSW) and HIV status (comparing HIV(+)MSM and HIV(-)MSM), rows correspond to individual genera and columns include log fold changes (“LFC”), p-values (“p”), false discovery rate adjusted p-values (“q”); LFCs correspond to change associated with HIV and MSM status

**Table S6: Biopsy Agrarian Microbiome Changes Stratified by Cohort.** Results of ANCOM-BC comparing biopsy microbiome genus abundance to time on the agrarian diet stratified by cohort, rows correspond to individual taxa and columns include log fold changes (“LFC”), p-values (“p”), false discovery rate adjusted p-values (“q”); LFCs correspond to change at later timepoints

**Table S7: Biopsy Agrarian Microbiome Changes.** Results of ANCOM-BC comparing biopsy microbiome genus abundance to time on the agrarian diet accounting for HIV and MSM status in the model, rows correspond to individual taxa and columns include log fold changes (“LFC”), p-values (“p”), false discovery rate adjusted p-values (“q”); LFCs correspond to change at later timepoints
